# Polycyclic Aromatic Hydrocarbons (PAHs) in aquatic ecosystem exposed to the 2020 Baghjan oil spill in upper Assam, India: Short-term toxicity and ecological risk assessment

**DOI:** 10.1371/journal.pone.0293601

**Published:** 2023-11-29

**Authors:** Vineet Singh, Ranjana Negi, Merin Jacob, Aaranya Gayathri, Anurag Rokade, Hiyashri Sarma, Jitul Kalita, Syeda Tabassum Tasfia, Rajendra Bharti, Abdul Wakid, Surindra Suthar, Vishnupriya Kolipakam, Qamar Qureshi

**Affiliations:** 1 Wildlife Institute of India, Chandrabani, Dehradun, Uttarakhand, India; 2 Assam Forest Department, Guwahati, India; 3 Aaranyak, Guwahati, Assam, India; 4 School of Environment & Natural Resources, Doon University, Dehradun, Uttarakhand, India; Fisheries College and Research Institute, INDIA

## Abstract

This study focuses on the short-term contamination and associated risks arising from the release of Polycyclic Aromatic Hydrocarbons (PAHs) due to the 2020 Baghjan oil blowout in upper Assam, India. Shortly after the Baghjan oil blowout, samples were collected from water, sediment, and fish species and examined for PAHs contents. The results of the analysis revealed ΣPAHs concentrations ranged between 0.21–691.31 μg L^-1^ (water); 37.6–395.8 μg Kg^-1^ (sediment); 104.3–7829.6 μg Kg^-1^ (fish). The prevalence of 3–4 ring low molecular weight PAHs compounds in water (87.17%), sediment (100%), and fish samples (93.17%) validate the petrogenic source of origin (oil spill). The geographic vicinity of the oil blowout is rich in wildlife; thus, leading to a significant mass mortality of several eco-sensitive species like fish, plants, microbes, reptiles, amphibians, birds and mammals including the Gangetic River dolphin. The initial ecological risk assessment suggested moderate to high-risk values (*RQ* >1) of majority PAHs concerning fish, daphnia, and algae species. This study highlights the need for recognizing the potential for short-term exposure to local species. To safeguard local ecosystems from potential future environmental disasters, it is imperative for the government to adopt a precautionary strategy.

## Introduction

Petroleum Products (oil and natural gas) are a complex mixture of hundreds of organic constituents, the vast majority of which are hydrocarbons. Among these, PAHs are one of the most common types of persistent organic pollutants (POPs) made up of two or higher aromatic rings of carbon and hydrogen atoms bonded in different configurations [1–3]. In the past few decades, numerous PAHs have undergone extensive research and some of the most extensively studied ones include Naphthalene, Acenaphthylene, Acenaphthene, Fluorene, Phenanthrene, Anthracene, Fluoranthene, Pyrene, Benz[a]anthracene, Chrysene, Benzo[b]fluoranthene, Benzo[k]fluoranthene, Benzo[a]pyrene, Dibenz[a,h]anthracene, Benzo[g,h,i]perylene and Indeno[1,2,3-cd]pyrene [4]. As a result, the European Union and U.S. Environmental Protection Agency have classified these 16 PAHs as high priority contaminants that require monitoring and quantification in all aquatic environments [4–7]. PAHs exhibit strong persistence as pollutants due to their high octanol/water partition coefficient (K_ow_), low water solubility, and strong attraction to lipophilic substances [8]. When PAHs infiltrate aquatic ecosystems, they have a tendency to attach themselves to suspended particulate matter, sediments, and organisms that inhabit these environments [9], posing ecotoxicological risks to various aquatic organisms. Additionally, PAHs have been scientifically proven to possess mutagenic and carcinogenic properties [10, 11].

These are one of the most marked toxic and ubiquitous semi-volatile organic pollutants in the environment generated naturally by the incomplete combustion or accidental burning of the forest and grassland, decaying organic matter and volcanic eruptions [12, 13]. In addition to natural origins, the primary anthropogenic causes like combustion engines, residential heating, industrial activities, coal gasification, petroleum refineries, oil spills, accidental discharge, stubble and biomass burning are the essential sources of PAHs in the environment [14–16]. Anthropogenic sources can be categorized into two groups based on where they come from: petrogenic and pyrolytic. Pyrolytic sources include the incomplete burning of engine oil and diesel fuel, coal, grass and forest fires, and fossil fuels. Petrogenic sources include petroleum products, crude oil discharge, and oil spills [17, 18]. The LMW-PAHs with 2 to 3 rings primarily stem from petrogenic sources, whereas the HMW-PAHs with 4, 5, and 6 rings typically originate from pyrolytic background [19–21].

Oil spill accidents are among the most severe events concerning petroleum pollution in aquatic environments [22–25]. In the past few decades, several massive oil spill catastrophes have been recorded worldwide, releasing massive amounts of crude oil and condensate into the marine ecosystems in the Gulf of Mexico [26–29]; Brazilian coast [30]; South Korean coast [31]; Gulf of Oman [32]; Coast of Hong Kong [31]; the coast of France [33, 34]. Although oil spills along the shore receive a lot of attention, but oil spills in standard and minor rivers may have a more detrimental impact on the environment due to their wide geographical distributions, low ability to dilute and spread, and interaction with waterborne particles and sediment [28, 35–37]. Additionally, the oil spills in rivers usually happen near inhabited areas, which makes them a bigger threat to public health because they could contaminate drinking water sources [38]. Nevertheless, the spilled crude oil spread across a large area of the ecosystem and severely impacted the freshwater and marine biota by causing direct toxicity and affecting their physiological functioning in the long run [28–30, 32]. Oil-originated PAHs easily bio-accumulates in the aquatic ecosystem and thus could be a possible cause of human food chain contaminations in such areas [29, 39, 40].

**Fig 1:**
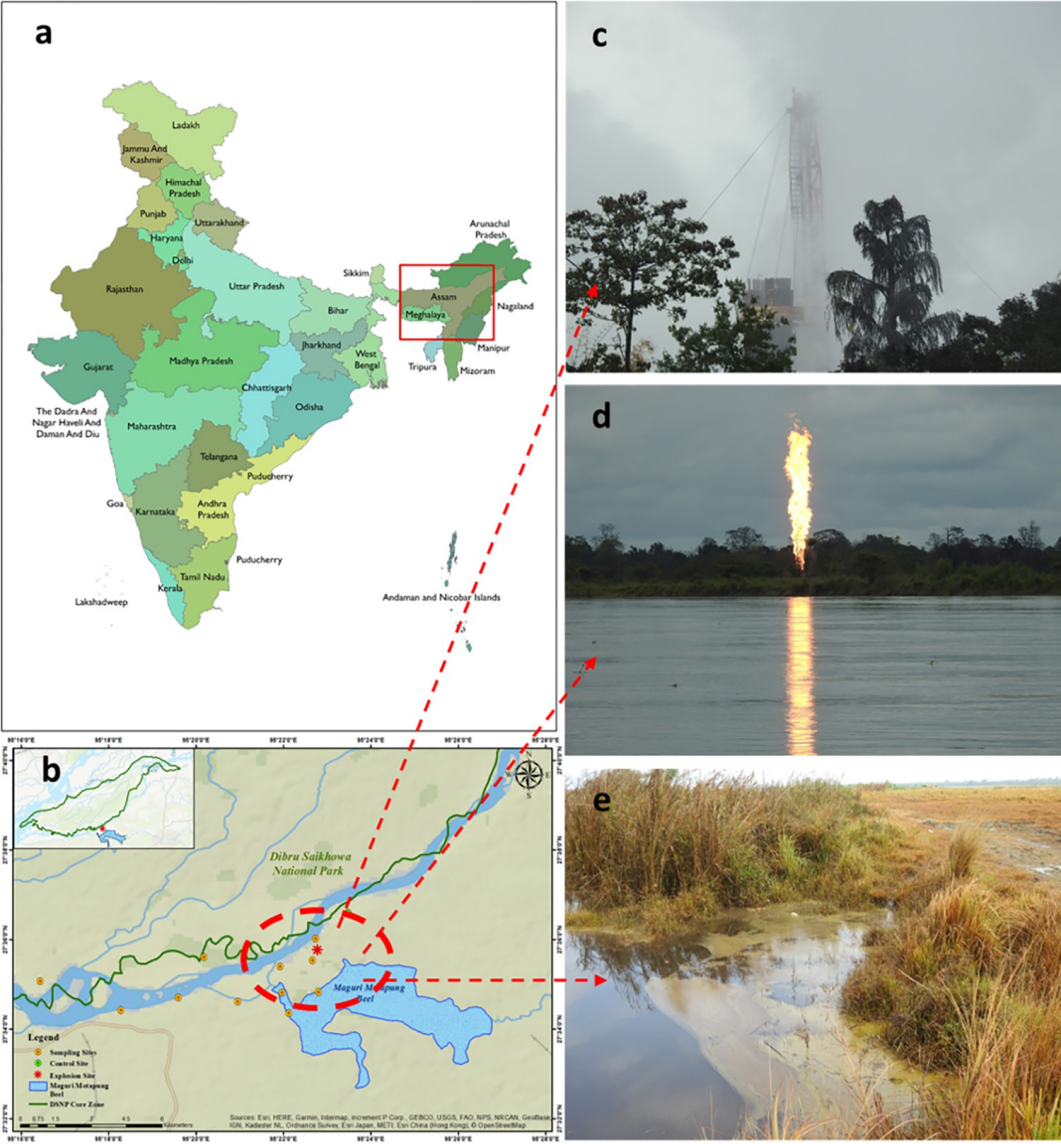
This is the Fig 1 (a) India Map (India Administrative Boundaries 2023, ESRI India), (b) Oil spill site (Source: Esri OpenStreetMap), (c) leakage of natural gas and condensate, (d) fire at blowout site, (e) oil contamination at Maguri Motapung Beel due to blowout.

Oil India Limited (OIL), a state-owned company, operates the Baghjan Oil Field in Upper Assam’s Tinsukia district. On May 27, 2020, a blowout from well Baghjan-5 took place there [41]. On the evening of the incident, there was beginning of oil and gas leakage from the well, which lasted for the next 14 days [42]. This discharge included the release of natural gas, condensation, and other toxic substances. Subsequently, on June 9^th^, 2020, ignition of the released oil resulted in a significant fire outbreak, leading into a blazing inferno. After catching fire, the oil well burned for the next 159 days. The blowout’s natural gas and condensate coated the surrounding environment in an oily film, resulting in three human deaths, large-scale local evacuations, and environmental damage to the impact zone and neighboring wildlife protected areas (Dibru Saikhowa National Park (DSNP)), rivers (Lohit and Dibru River), and wetlands (Maguri Motapung Beel (MMB)) [41, 42]. The DSNP is the 3^rd^ largest National Park in Assam, constituting Littoral and swamp forests, semi-evergreen forest, deciduous forest, and wet evergreen forest patches. The core zone of DSNP is the home of 38 species of Orchids. This protected area is known worldwide for its feral horses and diverse species, many of which are endangered or endemic [43–45]. This area is also recognized as a potential rhinoceros translocation site under the Indian Rhino Vision 2020 [46]. The Dibru River flows through the MMB via a unique system of channels that includes both lotic (flowing water) and lentic (standing water) ecosystems and is home to a diverse array of aquatic life. The Dibru River (DR) merges with the Lohit River (LR), a branch of the mighty Brahmaputra that flows between the park and the blowout area (Fig 1). These areas are also protected habitats and resting places for a significant population of the endangered Gangetic River dolphin (*Platanista gangetica*), which comes under Schedule-I species of Indian Wildlife (Protection) Act, 1972, found in the rivers, mainly in LR and Siang River surrounding DSNP [42].

In the immediate aftermath of the oil spill, samples were taken to determine the accumulation of PAHs in water, sediment, and local fish. The resulting data were then utilized to achieve two key objectives: (i) establishing the profile of PAHs composition and estimating their sources, and (ii) assessing the initial short-term ecological risk posed by the incident on local biota. This study aims to examine the impact of the early toxicity of the Baghjan oil spill on aquatic ecosystems through these comprehensive analyses.

## Material and methods

### Study site

The Baghjan-5 Oilwell (95° 22’ 51.066" E 27° 35’ 46.566" N) is located in Baghjan village of Tinsukia district in Assam, surrounded by 7–10 villages mainly habituated by local tribal communities. The area is surrounded by two major rivers Dibru and Lohit Rivers and a variety of other ecosystems, such as wetlands, swamp woods, and grasslands (Fig 1). The nearest area of environmental concern is the Maguri Motapung Beel (MMB) (10 sq. Km), a birdlife international designated Important Bird Area (IBA) [47]. A variety of ecologically important flora and fauna inhabit the nearby water bodies and surrounding grasslands. Most people in the MMB’s surrounding villages depend on fishing for a living, and nearly 95% of them are directly reliant on the wetland, which is part of the Dibru-Saikhowa Biosphere Reserve (DSBR). This area is approximately 100–200 meters away from the blowout site in the south and southeast direction. The blowout site is also adjoining DSNP, which is spread over 350 to 650 sq Km to the north, northeast, and northwest side of the blowout. The park’s closest point is about 900 meters away from the blast site.

### Reagent and chemicals

The analytical standards of 16 toxic PAHs compounds are listed on the World Health Organization’s (WHO) and United States Environmental Protection Agency’s (US EPA) lists of priority pollutants [48, 49], were obtained from the Sigma-Aldrich (USA). The physicochemical structures and other properties of selected PAHs compounds are described in S1 Table. The purities of the PAHs standard were in the ranges of 97.9–99.9%. Mixed standard solutions holding all the compound of interest were prepared by diluting the standard solution with Methanol (MeOH) and kept at -18°C in darkness to avoid photodegradation. For extraction and chromatographic analysis, HPLC grade Methanol (MeOH), Acetonitrile (ACN), and acetic acid (Merck, Germany) were used.

### Sample collection

**Fig 2:**
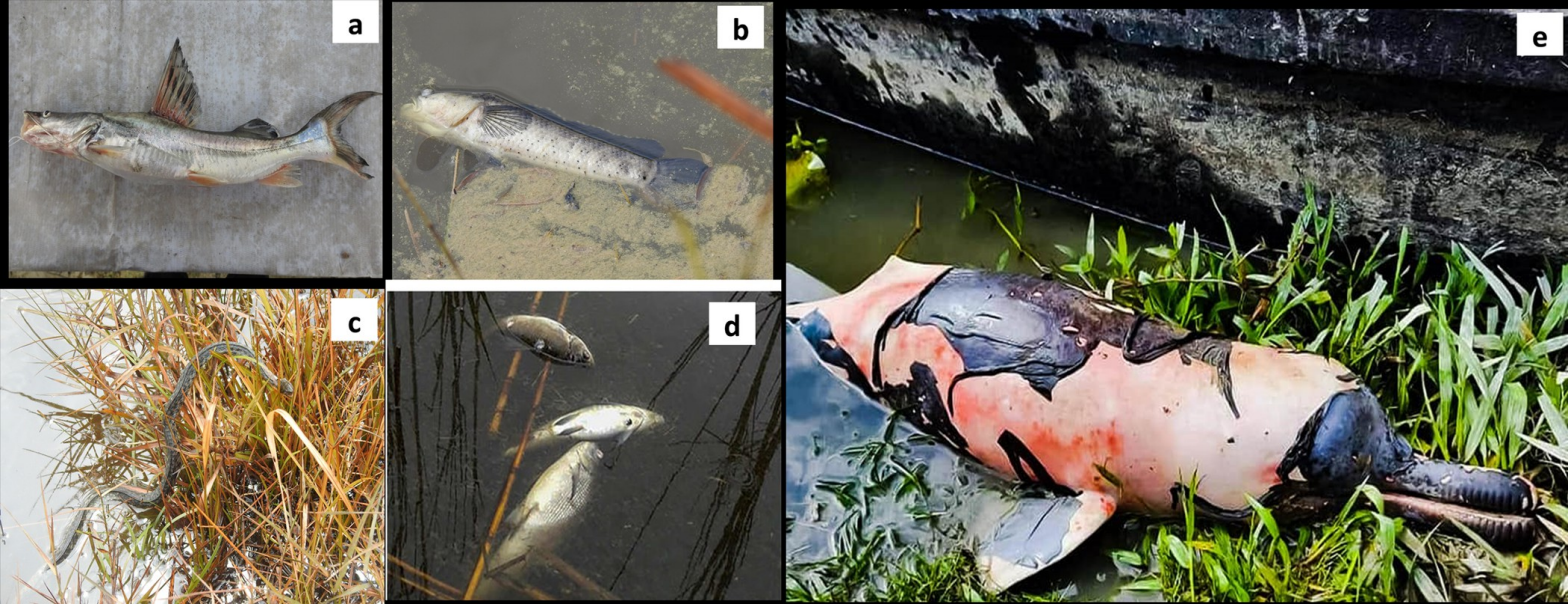
This is the Fig 2 Carcass of wild animals collected from the affected area (a) *S*. *seenghala*, (b) *Channa striata*, (c) snake (d) *Punitus sophore* (e) Gangetic River Dolphin (*Platanista gangetica*).

On May 27^th^, 2020, the oil well blowout at Baghjan oil fields caused a projected oil spill in a radius of 2 sq. km; there are anecdotal reports of a much bigger spread range [42]. The Wildlife Institute of India (WII) team arrived at the accidental site the same day and began systematic sampling, which lasted until June 3, 2020 (Fig 2). During this period, a total of 29 samples (water—12, sediments/soil—12 and fish—5) were collected from 12 different locations throughout the blowout impacted areas and control site to analyze the impact of the oil blowout. The details of the sampling location are provided in S2 Table. Based on the oil spill exposure, the samples were separated into three sampling areas, i.e., MMB, LR and DR. Water samples (2 L) were taken at each sampling station and placed in pre-cleaned sterile amber glass bottles. Waterbed sediments (200 g) were taken from the banks of the river and from the bottom of wetlands, where fine-textured substrate had gathered, and put in a sampling container. Five different species of dead fish (*Channa orientalis*, *Mystus Vittatus*, *Puntius Sophore*, *Rasbora daniconus*, and *Eutropiichthys vacha*) were taken from each of the two sites (MMB and LR). After sampling, all the samples were immediately stored in a cooling box (below 4°C) and transported to the laboratory for further analysis. The PAHs concentration in the water, sediment and fish tissues was analyzed using GC-MS. The physicochemical parameter, dissolved oxygen (DO) in water was also measured in situ using the Pro-DSS handheld multi-parameter probe (YSI, USA).

### Extraction of water samples

In water samples, PAHs were extracted according to the method by Chen et al. [50], with some modifications. The 1L water samples were extracted using Oasis Hydrophilic-Lipophilic Balance—Solid Phase Extraction (HLB-SPE) cartridges (6cc, 500 mg, Waters, Milford, MA, USA). Before extraction, the water samples were filtered and spiked with Deuterium labelled surrogate standard mixture solution (10 μL) (Naphthalene-D8, Acenaphthene-D10 and Phenanthrene-D10). Next, 6 ml of methanol and ultrapure water were then used to condition the HLB-SPE cartridges. The spiked water samples were then passed through the SPE columns at a flow rate of 5–10 mL min^-1^. After that, the SPE cartridges were cleaned with 6 mL Milli-*Q* water to eliminate the impurities and then a vacuum pump was used to dry the cartridges for 30 min. Later, 6 mL of ACN (2 × 3 mL) was added to the dried cartridges and allowed to soak for 5 min. Next, the cartridges soaked in ACN were eluted and collected in clean glass vials, concentrated to 0.5 mL, using a gentle nitrogen stream. Finally, the concentrates were filtered using a 0.22 μm syringe filter (PTFE, Cole Parmer, USA) and stored at—20°C in GC (amber) vials for further Gas Chromatograph-Mass Spectrometry (GC-MS) analysis.

#### Extraction of sediment and fish samples

Before the extraction, the sediment samples were dried, and the fish samples were properly eviscerated and filleted. Each sample was then homogenized using a mortar and kept frozen until extraction. The sediment and fish samples were extracted in the laboratory by the QuEChERS method described by Oduntan et al. [51], with some minor modifications. First, about 5 g of fish/sediment homogenized samples were added to the 50 mL QuEChERS tubes. Next, the samples were spiked with a surrogate standards mixture solution [52]. The spiked sediment/fish samples were hydrated by adding 3 mL ultra-pure water and allowed to stand for 1 hr. After that, 7 mL of ACN (mixed with 1% acetic acid) was added to the tube and vortexed for 1 min (Remi VM-100, Mumbai, India). This step was followed by the addition of 2.5 g of anhydrous MgSO4 (facilitate solvent partition) and 1 g of C_2_H_3_NaO_2_ (buffer) into the tube and ultrasonicated for 5 min for better extraction and then centrifuged (7000 rpm). After centrifugation, the aliquots were moved to a 2 mL d-SPE tube comprising 150 mg anhydrous MgSO_4_ and 25 mg C18 (to remove access fat) for clean-up. The samples were vortexed for 1 minute and centrifuged for 5 minutes at 4000 rpm. After centrifugation, the final supernatant was filtered using a 0.45 μm PTFE syringe filter (Cole Parmer, USA). Finally, the samples were injected into the GC-MS for analysis.

### Instrumental and analytical conditions

PAHs were determined using a Shimadzu TQ 8040 with a Triple Quadrupole Gas Chromatograph-Mass Spectrometry (GC-MS, Kyoto, Japan) with an auto-injector. The target analytes were separated using a Restek Rtx-5MS Column (non-polar column, 5% -phenyl methylpolysiloxane 60 m length x 0.25 mm inner diameter x 0.25 μm film thickness). Helium (99.99% purity) was used as a carrier gas, and the flow rate was held constant at 1.5 mL min^-1^. The injection port temperature was held at 270°C, and a splitless injection of 1 μL was injected using an auto-sampler with a 150 kPa internal pressure. The oven temperature was maintained at a gradient mode following the initial column temperature at 70°C, held for 1 minute and ramped from 70°C to 150°C at the rate of 20°C min^-1^, held for 2 minutes, and finally to 300°C at 5°C min^-1^ and held for 20 minutes. The total run time was 55 min. The GC interface temperature was maintained at 280°C. The electron impact ionization (EI) mode at 70 eV was used for the GC-MS study. The ion source was set to 230°C. The details of calibration curves and details of ions of the PAHs are provided in S3 and S4 Tables. For MS detection, the scan technique was used, with m/z values ranging from 35 to 500. The PAH speak identifications were made with the aid of the NIST mass library.

### Quality control

The 16 PAHs were identified and quantified by comparing the retention time with a certified standard solution to ensure the analytical method’s integrity and performance. Before instrumental analyses for quantification, 320 ng/g deuterated PAH internal standards were applied to all the extracted samples. The matrix recoveries were calculated by testing appropriate certified reference material (CRM). The CRM (IAEA-408 sediment) was used to ensure the accuracy of the PAHs measurements in sediment samples, and ’IAEA-435’ Tuna homogenate was used to determine the quality assurance of PAHs in fish samples. For both reference materials, the measurement precision was within 5% of the approved values. For the analytical recovery of water samples, spiked PAHs samples were used. The average recoveries of PAH-spiked water samples collected from GC–MS analysis ranged from 98.9 to 111%, and the limit of detection (LOD) and limit of quantification (LOQ) for individual PAHs in all the matrices ranged from 0.02 and 0.12 μg/L with a signal-to-noise ratio of 3 and a limit of quantization of signal-to-noise ratio of 10. No targeted PPCPs of interest were found in the field blank samples, suggesting that there was no major contamination during analysis, extraction, or sampling.

### Ecological risk assessment

The collaborative method to assessing the effects of chemical exposure on habitats is known as risk assessment. The Risk Quotient method is a widely accepted method for conducting risk assessment analysis [53]. The risk quotient (RQ) is defined as the proportion of MEC to PNEC (Eq 1):

$$RQ=\frac{MEC}{PNEC}$$ [Eq 1]

Where, MEC is the maximum measured environmental concentration and PNEC is the predicted no-effect concentration.

### Descriptive analysis

The data analysis was conducted using the Statistical Package for the Social Sciences (SPSS) 21, a well-established software extensively utilized for data visualization and statistical analysis. The graphs were crafted using this software and Microsoft Excel, both employed to ensure the precision and comprehensiveness of data representation and analysis. The final findings are presented as an average ± SD for each sample obtained from various sampling points.

## Results and discussion

### PAHs in water

**Fig 3:**
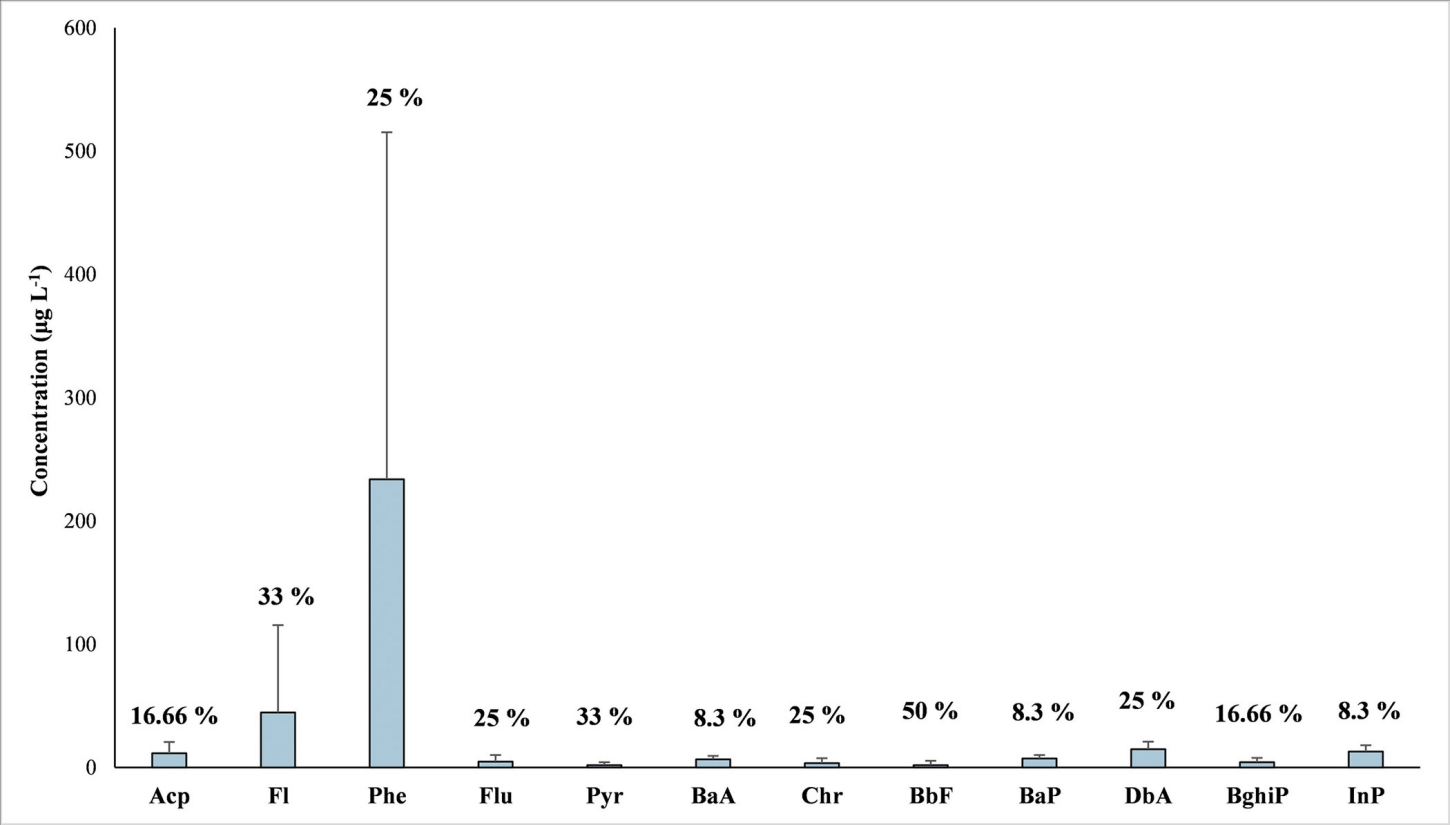
This is the Fig 3 the average individual concentration of detected PAHs in the collected water samples with detection frequencies (%).

The concentration range, average value and detection frequencies (%) of sixteen targeted PAHs in the water, sediments, and fish tissue samples collected from the oil blowout-affected areas are presented in Table 1. Out of 16 PAHs, the highest distribution was recorded for MMB (12 PAHs), followed by DR (7 PAHs) and the lowest in the LR (1 PAHs). Among the detected PAHs, BbF was present in 50% of water samples. The overall concentration and detection frequencies (%) of individual PAHs in water samples are mentioned in Fig 3. The total PAHs in water extended from 0.21 to 691.31 μg L^-1^, with an average value of 32.11 μg L^-1^. The range of overall PAHs (ΣPAHs) was monitored highest in the MMB (0.21–691.31 μg L^-1^) trailed by the DR (0.24–7.28 μg L^-1^) and LR (0.22–0.29 μg L^-1^) with average concentrations of 46.88, 1.45 and 0.26 μg L^-1^, respectively. The concentration of PAHs in water samples varied significantly between places due to OIL’s extensive use of water to extinguish the blowout and fire. This enormous volume of water was exposed to blowout gases, and condensate was drained directly into the MMB, DR, and LR without treatment.

**Table 1:** This is the Table 1 the descriptive analysis of PAHs concentration in the collected water and fish samples.

PAHs	Water (μg L^-1^)		Fish (μg Kg^-1^)	
	Range	Avg ± SD	Range	Avg ± SD
**Nap**	-	-	104.3–166.9	135.6 ± 44.26
**Acpy**	-	-	ND—129.6	129.6
**Acp**	0.26–22.62	11.44 ± 15.81	475.5–1500.9	841 ± 463.68
**Fl**	0.21–174.6	44.55 ± 86.70	2779.4–7829.6	4142.67 ± 2462.86
**Phe**	2.8–691.31	233.79 ± 396.22	200.1–7770	5609.7 ± 3620.19
**AnT**	-	-	-	-
**Flu**	0.42–13.33	4.73 ± 7.44	178.9–347.4	263.15 ± 119.14
**Pyr**	0.23–6.12	1.80 ± 2.88	136.5–317.7	227.1 ± 128.12
**BaA**	ND—6.61	6.61	145.1–169.3	157.2 ± 17.11
**Chr**	0.36–9.97	3.57 ± 5.54	135–240.4	187.7 ± 74.52
**BbF**	0.27–9.18	1.81 ± 3.61	-	-
**BkF**	-	-	-	-
**BaP**	ND—7.17	7.17	-	-
**DbA**	ND—14.82	14.82	-	-
**BGP**	0.24–8.34	4.29 ± 5.72	-	-
**InP**	ND—12.82	12.82	-	-

PAHs such as Acp (0.26–22.62 μg L^-1^), Fl (0.21–174.6 μg L^-1^), Phe (2.8–691.31 μg L^-1^), Flu (0.42–13.33 μg L^-1^), Pyr (0.23–6.12 μg L^-1^) and Chr (0.36–9.97 μg L^-1^) were found at high concentrations in the water samples. The seven carcinogenic PAHs (ΣCPAHs), including BbF, BkF, BaA, Chr, BaP, DbA and InP were also found in water samples ranging from 0.27 to 14.82 μg L^-1^, with an average of 4.83 μg L^-1^, accounting for 6.33% of total PAHs in all the water samples. The highest ΣCPAHs concentration was reported in MMB (0.27–14.82 μg L^-1^) followed by DR (0.36–0.52 μg L^-1^) and LR (0.27–0.29 μg L^-1^). Benzo[a]pyrene (BaA), assigned as the most carcinogenic pollutant of all PAHs [31], was detected in a single water sample collected from the MMB. The findings were also compared with the published literature on PAHs concentrations in surface water. The current study’s PAHs concentrations were found to be significantly higher than those observed in Indian surface water, Gomti river (0.65–75.57 μg L^-1^) [54], Brahmaputra river (ND– 31 μg L^-1^) [55, 56], Hooghly river (ND– 31 μg L^-1^) [56] and groundwater study ranging from ND– 143.2 μg L^-1^ [57] and other parts of the world like Mississippi river (12–430 ng L^-1^) [58], ten river in Tianjin industrial complex area in China (45.81–1272 ng L^-1^) [59], Pearl river delta, China (55.5–522 ng L^-1^) [60].

PAHs enter the aquatic environment primarily via an oil spill, riverine drainage, and urban and industrial effluents [29, 30, 61, 62]. The 2–3 ringed low molecular weight (LMW) PAHs are more likely to persist in the water after being released, where they can be ingested or breathed in by aquatic organisms [29, 30]. The high molecular weight (HMW) PAHs stay in sediments and particulate matter because they don’t get photochemically oxidized or biologically broken down [63, 64]. When environmental conditions change as a result of hydrological processes such as waves and currents, seasonal variations, as well as sand mining, dredging and boat traffic, such contaminants are released back into the water, causing secondary pollution and long-term toxicity affecting all life forms including humans [29, 40, 65–67].

### PAHs concentration in sediment samples

Out of 12 samples, only three PAHs compounds were found in a single sediment sample (S-9) collected from the oil blowout’s maximum impacted region, i.e., MMB. The PAHs concentration in the sample ranged from 37.6–395.8 μg Kg^-1^, with an average of 195.23 μg Kg^-1^. The sediment samples were contaminated with three-ring PAHs compounds (Acp, Fl, and Phe). The absence of PAH detection in other sediment samples could be explained by the fact that oil on the water’s surface takes time to sink to the bottom floor following a series of chemical and physical changes (including weathering, evaporation, oxidation, biodegradation, and emulsification) that cause spilled oil to breakdown and become heavier than water [29, 30]. This is a time-consuming process, and the studied samples were collected immediately after the oil blowout. The PAHs concentration in the MMB sediment sample appears to be in the middle of the range when compared to sediment samples from other parts of India, including Bhavnagar coast (5.02–981.18 μg g^-1^) [43], Brahmaputra river (6–798 ng g^-1^) [56], Hooghly river (48–1329 ng g^-1^) [56], Sundarbans (208.3–12993.1 ng g^-1^) [68], and in the world including Chaohu river and Taihu lake, China (36.5–2530 ng g^-1^) [69, 70], Anzali wetland, Iran (212–2674 ng g^-1^) [71], Todos-Santos Bay wetland, Mexico (ND– 96 ng g^-1^) [72] and Potos Lagoon, Brazil (7.3–92.8 μg Kg^-1^) [73].

### Concentration of PAHs in fish samples

Table 1 shows the amount of each PAH found in different fish species taken from the MMB and LR. Nine PAHs were found in the samples with substantially higher concentrations, ranging from 104.3 to 7829.6 μg Kg^-1^, with an average value of 1932.37 μg Kg^-1^. The concentration of ΣPAHs was highest in the fish samples from the MMB. The most water-soluble and widely available LMW PAHs were predominant in all the samples. Acp, Fl, and Phe accounted for greater than 95% of the total detected PAHs concentration, while the high molecular weight (HMW) PAHs were negligible. The ΣPAHs (sum of 16 PAHs) in the fish species were recorded highest in *Mystus vittatus* (11467.9 μg Kg^-1^), followed by *Channa orientalis* (11378.1 μg Kg^-1^), *Rasbora daniconius* (10877.7 μg Kg^-1^), and *Puntius sophore* (10721.0 μg Kg^-1^). Fish samples from the heavily damaged MMB and DR area have much greater PAHs concentrations than the water samples from the exact location, because of the fishes’ higher bioaccumulation efficiency [29, 40, 74, 75].

**Fig 4:**
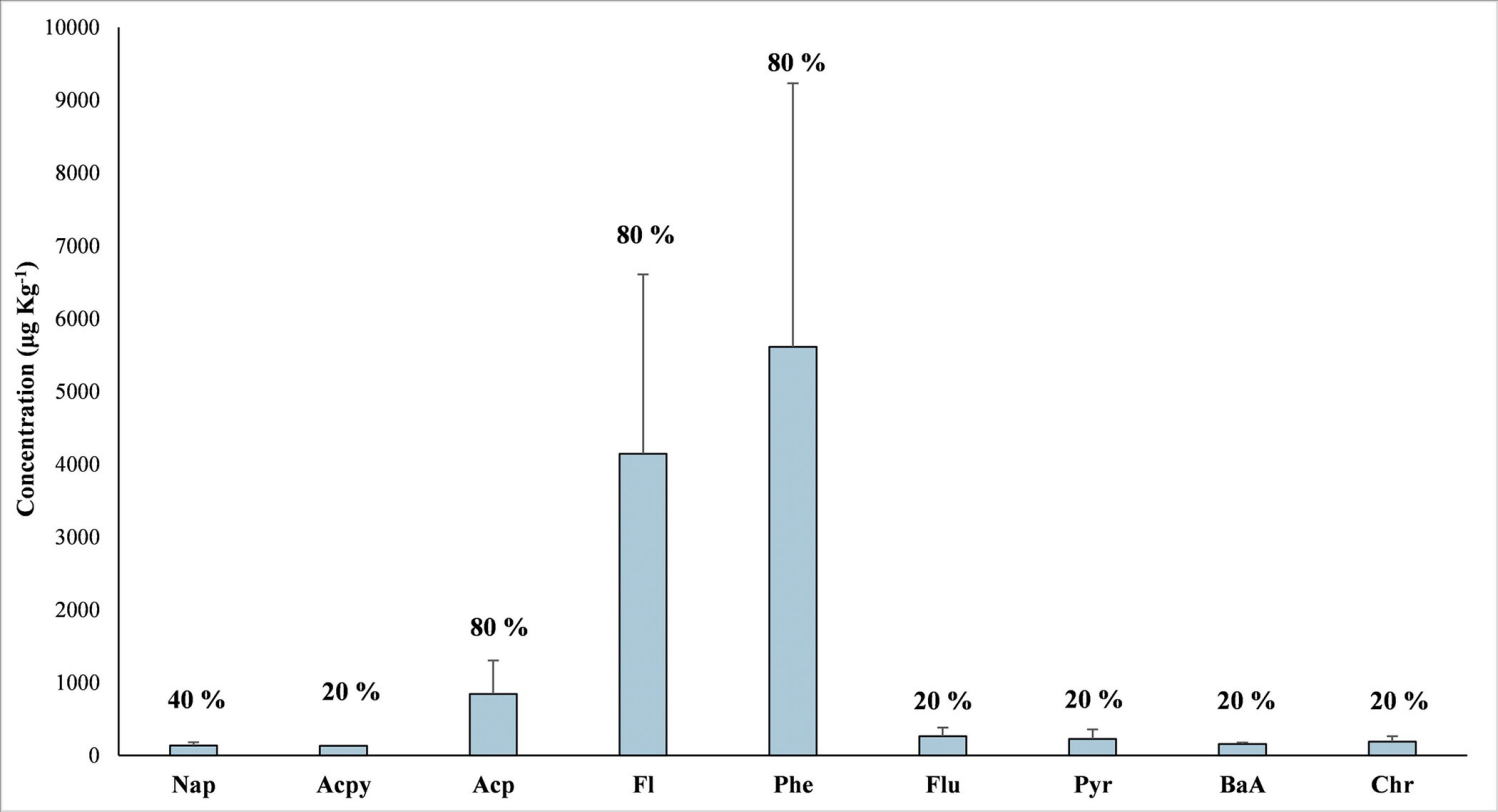
This is the Fig 4 the average individual concentration of detected PAHs in the collected fish samples with detection frequencies (%).

The fish sample (*Eutropiichthys vacha)* collected from the control site (upstream of LR) showed no PAHs concentration. The overall concentration and detection frequencies (%) of individual PAHs in fish samples are mentioned in Fig 4. The carcinogenic PAHs (BaA and Chr) were also found in fish samples ranging from 135.0 to 240.4 μg Kg^-1^, with an average of 172.5 μg Kg^-1^, accounting for 1.55% of total PAHs in all the fish samples. The ΣPAHs concentration in fish samples collected from oil-affected MMB appears to be 10 to 100 times higher than previously reported concentrations in India including fish samples from Gomti river (12.85–34.89 ng g^-1^) [76], Mumbai (17.43–70.44 ng g^-1^) [77], and other parts of the world like Nigeria (0.1–458.1 μg Kg^-1^) [78], Arabian Gulf (30–247 ng g^-1^) [79], Hong Kong (15.5–118 ng g^-1^) [80].

This blow-out caused mortality in several fishes, migratory birds, reptiles, livestock, and endangered Ganges River dolphin (Fig 2). Several fish were found dead in MMB due to a lack of oxygen and oil deposition in gills, resulting in asphyxiation and death of aquatic creatures due to hypoxia; moreover, sunlight penetration is also hindered, resulting in aquatic vegetation mortality [42, 81]. The presence of these PAHs in the aquatic ecosystem raises significant concern due to their specific toxicity. They induce a variety of biological dysfunctions, including neoplasms, compromised reproductive results and endocrine disruption leading to immunotoxicity. Additionally, these PAHs are associated with obstacles in post-larval growth and trans-generative impacts. Recent studies also indicate that extremely low levels of PAH seriously influence the development of fish [29, 40]. Thus, the PAHs concentrations found in this study will have severe toxic effects on fish, benthic organisms, and other aquatic animals [29, 32]. It has also been shown that PAHs can cause cancer and mutations, and that they can weaken the immune systems of fish and other aquatic fauna. Effects on immune system development, humoral immunity and host resistance have been well documented [61, 82–84].

Flooding, a natural event in the Brahmaputra River system, hugely impacted fish diversity by spreading these waters, densely packed with condensate and other harmful chemicals, across the entire landscape, causing severe harm to fish biota and their habitats. Oil slicks obstruct the exchange of gases between aquatic animals and the atmosphere [85, 86]. As a result, the dissolved gas circulatory equilibrium in water is compromised. Besides the measured dissolved oxygen (DO) recorded from the blowout site (0.93 ± 0.30 mg L^-1^), the MMB had the lowest DO concentration (1.06 ± 0.70 mg L^-1^) in this study, which could be related to the oil slick that forms on the surface of the MMB and limits oxygen mixing and aeration of the water. Further information on DO concentration at other sampling sites can be found in S5 Table.

### PAHs composition profile and source estimation

**Fig 5:**
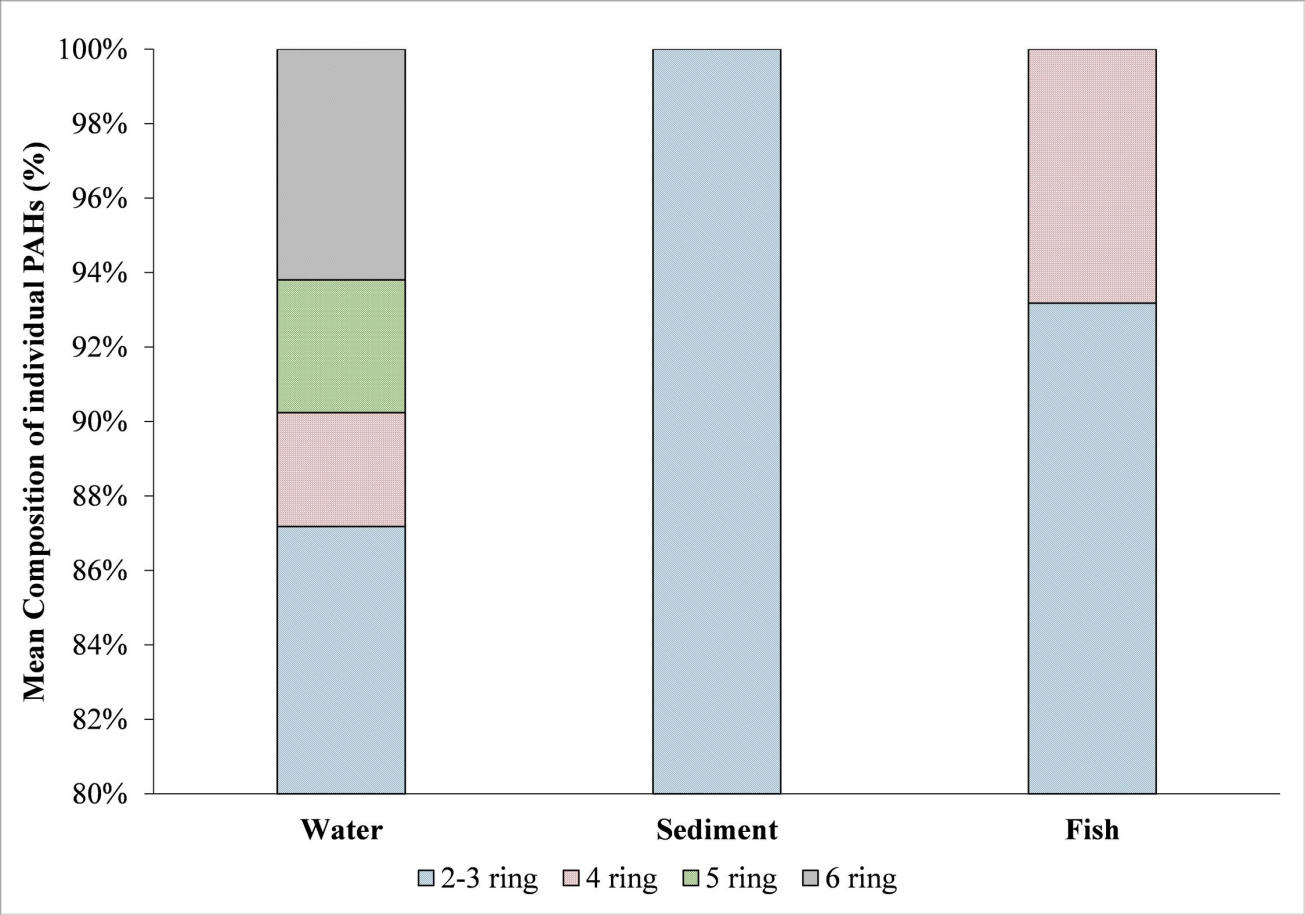
This is the Fig 5 PAHs composition profile in the collected water, sediment and fish samples from the oil blowout affected area.

PAHs in the environment come from two main places: natural emissions and anthropogenic activity [1–3]. This study shows the average relative distribution of all the detected PAHs in MMB, DR, and LR samples in Fig 5. The 2–3 ring LMW-PAHs were the most prominent in all the sampled matrixes. In water samples, the average per cent of LMW-PAHs was reported to be the highest (87.17%), and very nominal concentrations of HMW-PAHs were detected (12.81%). The single detected sediment sample from the MMB also showed 100% LMW-PAHs. Similar to water and sediment samples, the percentage of LMW-PAHs (93.17%) was also more dominant in the fish samples than the HMW-PAHs (6.82%).

**Fig 6:**
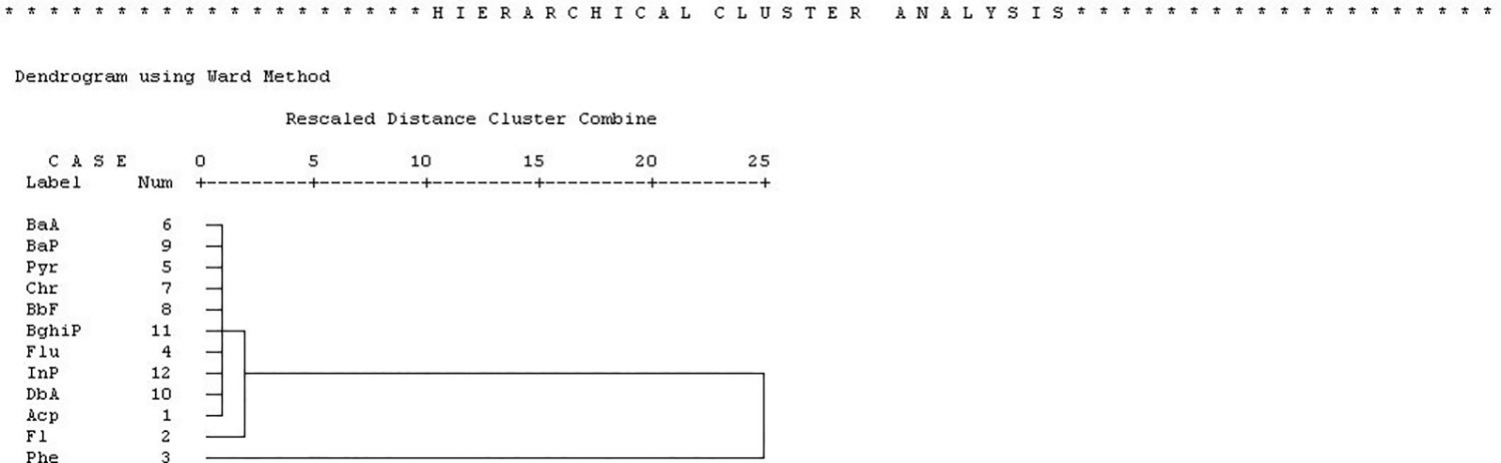
This is the Fig 6 the hierarchical dendrogram for 12 PAHs in the collected water samples using average linkage between groups.

Cluster analysis was also performed on the water samples to identify homogenous groups of individual PAHs. The hierarchical dendrogram generated by cluster analysis splits the 12 detected individuals’ PAHs into two distinct groupings (Fig 6). The first group is made up of Phe (LMW-PAHs), which are typically found in petrogenic sources. The second group is further subdivided into two more subgroups. The first category consists of Fl (another LMW-PAH), whereas the second subgroup consists of Acp (LMW-PAHs) and other HMW-PAHs from pyrogenic and petrogenic mix sources. The results demonstrate that the first leading group (Phe) and the first category of the second leading group (Fl) indicate the dominance of LMW-PAHs in the oil-affected area.

The detection of high concentration of LMW-PAHs in the studied area recommends a relatively recent local PAHs source that entered the aquatic ecosystem due to the oil blowout. The moderate to high lipophilicity of LMW-PAHs (log *K*ow 3.3 to *>*5.6) in the aquatic environment makes them readily available for absorption, adsorption and ingestion by fish and other aquatic organisms [29, 32, 87, 88]. Thus, over time, the bioaccumulation of these LMW PAHs in the aquatic organism tends to increase in trophic levels, causing PAHs to reach the threshold levels that may cause toxicity in the exposed aquatic organisms [89–91].

### Ecological risk assessment

**Fig 7:**
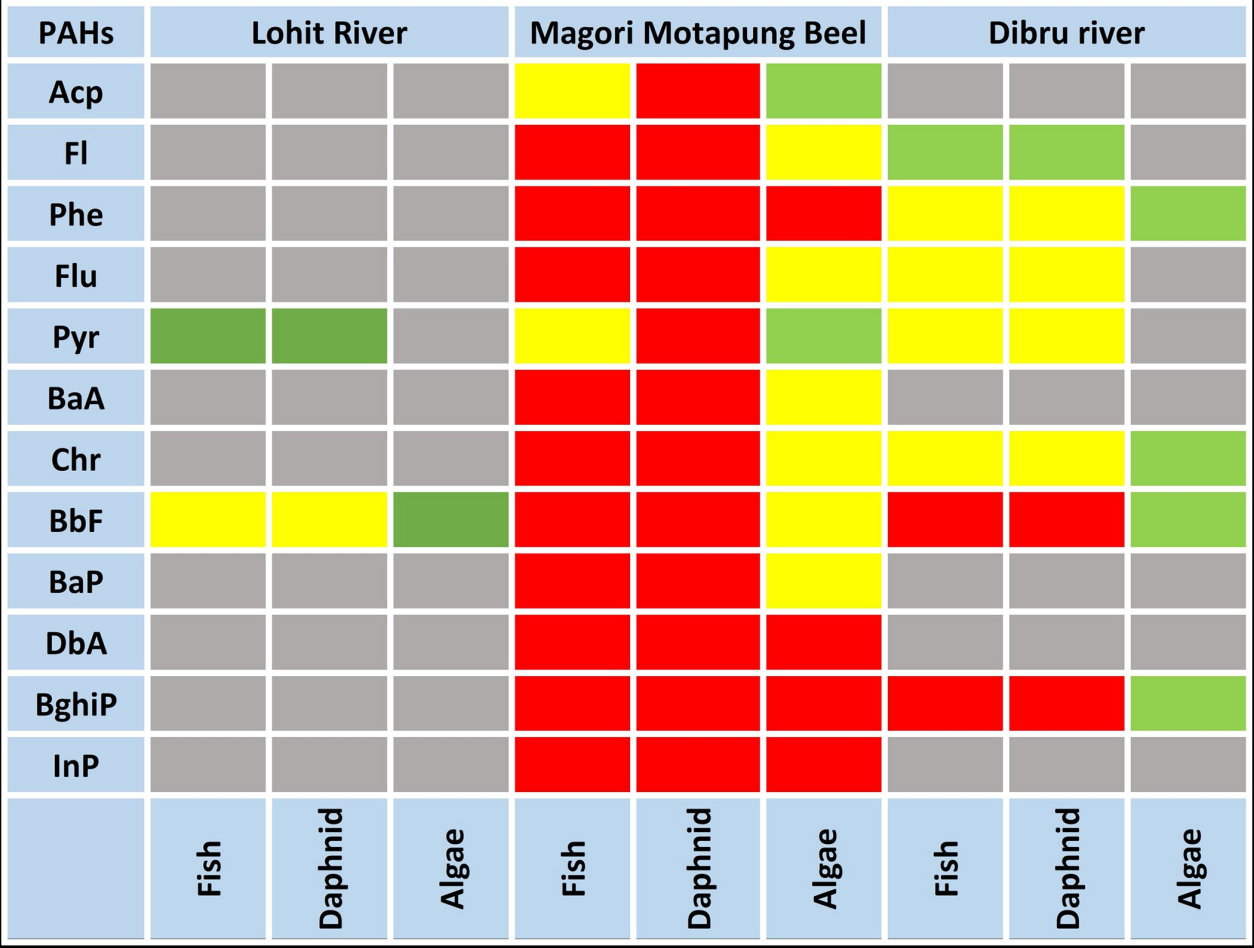
This is the Fig 7 ecological health risk assessment in aquatic biota calculated based on PAHs load in the water. RQ <0.01 (Gray) no ecological risk, 0.01≤ RQ <0.1 (Green) low risks, 0.1 ≤RQ <1 (Yellow) median risk, and RQ ≥1 (Red) high ecological risk Fl, Phe, Flu, BaA, Chr, BbF, BaP, DbA, BghiP and InP.

The oil-blowout disaster significantly impacted the area, surrounded by conservation zones, rivers, wetlands, and Important Bird Areas, all of which are crucial to the biodiversity and the livelihoods of residents. As the event occurred during a monsoon, the impact of hazardous condensate and chemicals spreading through air and water extends beyond the usual area of influence, causing widespread harm. PAHs are complex chemical compounds that have lethal impacts on flora and fauna [92]. The ecotoxicological risk quotient values related with exposure to individual PAHs for three characteristic trophic levels, viz., algae, aquatic invertebrates (*Daphnia*), and fish are estimated and described in Fig 7. The mean detected concentrations of PAHs in water at each sampling location were used to determine the Risk Quotient (*RQ*) scenarios to identify the high-risk sectors. Compounds with a Risk Quotient (*RQ)* < 0.01 pose no ecological risk; those with 0.01 < *RQ* < 0.1 pose a minimal risk; those with 0.1 < *RQ* < 1 pose a moderate risk; and those with *RQ* > 1 pose a severe ecological risk to the environment. The 12 PAHs found in the MMB (Fl, BaA, Phe, Flu, BaP, Chr, BbF, DbA, InP and BghiP) exhibited a high *RQ* >1 value for fish, *Daphnia*, and algae, indicating a significant toxicity burden in the aquatic organisms of the MMB. The HMW-PAHs (BbF and Bghip) demonstrated possible chronic and median hazards (0.1 < *RQ* < 1) for the examined aquatic organism in DR. Aside from that, Phe, Flu, Pyr, and Chr exhibited moderate toxicity to fish and micro-invertebrates. The LR showed the least toxicity compared to MMB and DR. The result signifies that the MMB and the adjacent aquatic ecosystem (DR) were detrimentally exposed to PAHs contamination released from the blowout and are vulnerable due to their unique hydrogeological structure as a wetland system.

Previous research show PAHs often concentrate near deposition sites in aquatic ecosystems. Relevant literature on PAHs uptake, retention and transport by aquatic species suggests that many aquatic animals rapidly acquire (bioconcentrate) PAHs from low ambient level [29, 32, 93].

### Effects on Gangetic river dolphin

Oil also has a wide range of effects on aquatic mammals, and it can be absorbed through various pathways, including cutaneous contact, inhalation, and ingestion [49, 94, 95]. Aquatic mammals frequently pass through the air-water interface because they are air-breathing organisms that get the majority of their food from below the surface. During this process, they get physically exposed and inhale volatilized oil [96]. Earlier research on the condition of cetaceans (Whales, Dolphins, and Porpoises) exposed to oil spills showed lung injuries and physical damage to the breathing system caused by stimulating tissues/membranes during inhalation of liquid oil collected on the blowhole [96, 97]. As a result, moderate to severe lung illnesses developed, resulting in lung abscesses, pneumonia, adrenal toxicity and pulmonary infections [96, 98]. Similar health effects, including adrenal and lung disorders, were found in dolphins that became stranded and died inside the oil spill in a study conducted by De Guise et al. [99] on dolphins exposed to oil in Barataria Bay, Louisiana [100, 101]. Further, when aquatic mammals consume prey containing oil or its metabolites or ingest contaminated water and sediment due to foraging, the likelihood of oil bioaccumulation increases [102, 103]. Therefore, freshwater mammals feeding and resting in a wetland are more susceptible to oil exposure and inhalation [104, 105].

The findings of this study supported the theory that oil spill exposure led Dolphins to develop adrenal and lung disease, contributing to the reported increase in dolphin mortality. The autopsy report of the Gangetic River dolphin body found in the MMB matched with prior reports of PAHs effects on mammals. Extensive hemorrhages in the gastrointestinal system and stomach, edema and hemorrhages in the lungs, hemorrhages and ventricular damage in the heart, liver parenchyma, intestinal lumen, kidney and brain congestion were among the main findings. The post-mortem examination revealed that the dolphin died as a result of inhaling or ingesting a hazardous chemical, which induced hypoxia [42].

## Conclusion

In conclusion, this study revealed the initial hazardous affect caused by the Baghjan oil blowout in the surrounding area. A significant increase in PAHs concentration within water and fish samples collected from the blowout and surrounding sites were observed. The conclusion obtained from this study suggested that:

1. In MMB wetland, the highest distribution of individual PAHs was recorded in the fish (104.3–7829.6 μg Kg^-1^), water (0.21–691.31 μg L^-1^), and sediment (37.6–395.8 μg Kg^-1^), samples, surpassing the DR and LR sites. The higher concentration in the MMB wetland can be attributed to the fact that wetland characterize low energy surrounding with minimum turbulent mixing. As a result, there is a reduction in the number of physical processes like dilution and washing out, as well as biodegradation losses of organic pollutants such as sewage and crude oil [106, 107]. Hence, make these environments become particularly susceptible to high accumulation of these pollutants [108]. This finding emphasizes the severe impact of the blowout on MMB.
2. The detected PAHs were predominantly petrogenic in origin, with 2–3 ring LMW PAHs accounting for the majority in collected water (87.17%), sediment (100%), and fish samples (93.17%). This composition suggests a natural petroleum products origin, likely crude oil discharge and oil spills.
3. The initial ecological risk assessment revealed elevated risk associated with LMW PAHs (*RQ* >1) for aquatic fauna, significant toxicity burden in the MMB and DR. The distressing discovery of dead Dolphin, multiple dead fishes, and numerous herpetofauna and insect species with burn injuries corroborates the toxic impact of PAHs. In addition, several plant species have died or wilted due to the oil spill, and the forest and grassland ecosystem has been seriously impacted.

The implication of this blowout extends beyond the immediate environment. The release of hazardous and toxic substances jeopardizes various form of life in general. The toxicity persists, posing long term threats to aquatic and soil systems, as well as human and wildlife. The blowout also negatively impacted the native population in neighboring communities as most people rely on fishing for a living.

### Future perspectives

A comprehensive, long-term monitoring is imperative to fully comprehend blowout’s ecological repercussions, considering the potential for bioaccumulation and entry of the PAHs into the food chain. The PAHs concentration in the studied areas may lead to moderate (DR and LR) to high (MMB) risks for the aquatic ecosystem. Of particular concern are the highest detected PAHs (Acp, Fl, and Phe) in water, sediment, and fish, as they may imperil bottom dwelling organisms. Ultimately, these pollutants could concentrate in the sediment and soil, becoming integral components of the overall ecosystem, interacting with the flora, fauna, and humans. This study underscores the urgency for stringent measures in dealing with such blowouts. Without effective intervention, the devastating consequences of such incidents are inevitable. The importance of safeguarding against and mitigating the aftermath of such blowouts cannot be overstated.

## Supporting information

S1 Table The chemical structures of PAHs compounds and their physicochemical properties. (DOCX)

S2 Table Details of sampling locations. (DOCX)

S3 Table Regression equations and coefficients of determination (R2) obtained for studied PAHs. (DOCX)

S4 Table Molecular weights, quantification ion, confirmation ions and retention times of studied PAHs. (DOCX)

S5 Table Dissolved Oxygen concentration at sampling sites. (DOCX)

## References

[1] Neff JM, Stout SA, Gunster DG. Ecological risk assessment of polycyclic aromatic hydrocarbons in sediments: Identifying sources and ecological hazard. Integr Environ Assess Manag. 2005;1: 22–33. doi: 10.1897/ieam_2004a-016.1 16637144

[2] Slezakova K, Pires JCM, Castro D, Alvim-Ferraz MCM, Delerue-Matos C, Morais S, et al. PAH air pollution at a Portuguese urban area: carcinogenic risks and sources identification. Environ Sci Pollut Res Int. 2013;20: 3932–3945. doi: 10.1007/s11356-012-1300-7 23184127

[3] Nakata H, Uehara K, Goto Y, Fukumura M, Shimasaki H, Takikawa K, et al. Polycyclic aromatic hydrocarbons in oysters and sediments from the Yatsushiro Sea, Japan: comparison of potential risks among PAHs, dioxins and dioxin-like compounds in benthic organisms. Ecotoxicol Environ Saf. 2014;99: 61–68. doi: 10.1016/j.ecoenv.2013.10.005 24211160

[4] Ćirić S, Mitić V, Jovanović S, Ilić M, Nikolić J, Stojanović G, et al. Dispersive micro-solid phase extraction of 16 priority polycyclic aromatic hydrocarbons from water by using thermally treated clinoptilolite, and their quantification by GC-MS. Microchimica Acta. 2018. doi: 10.1007/s00604-018-3091-0 30465108

[5] Munyengabe A, Ndibewu PP, Sibali LL, Ngobeni P. Polymeric nanocomposite materials for photocatalytic detoxification of polycyclic aromatic hydrocarbons in aquatic environments-A review. Results Eng. 2022;15: 100530. doi: 10.1016/j.rineng.2022.100530

[6] Nahar A, Akbor MA, Sarker S, Bakar Siddique MA, Shaikh MAA, Chowdhury NJ, et al. Dissemination and risk assessment of polycyclic aromatic hydrocarbons (PAHs) in water and sediment of Buriganga and Dhaleswari rivers of Dhaka, Bangladesh. Heliyon. 2023;9: e18465. doi: 10.1016/j.heliyon.2023.e18465 37560670 PMC10407051

[7] Hussar E, Richards S, Lin ZQ, Dixon RP, Johnson KA. Human health risk assessment of 16 priority polycyclic aromatic hydrocarbons in soils of chattanooga, Tennessee, USA. Water Air Soil Pollut. 2012;223: 5535–5548. doi: 10.1007/s11270-012-1265-7 23243323 PMC3521527

[8] Rizzi C, Villa S, Waichman A V., de Souza Nunes GS, de Oliveira R, Vighi M, et al. Occurrence, sources, and ecological risks of polycyclic aromatic hydrocarbons (PAHs) in the Amazon river. Chemosphere. 2023;336: 139285. doi: 10.1016/j.chemosphere.2023.139285 37353170

[9] Montuori P, De Rosa E, Duca F Di, Simone B De, Scippa S Russo I, et al. Polycyclic Aromatic Hydrocarbons (PAHs) in thDissolved Phase, Particulate Matter, and Sediment of the Sele River, Southern Italy: A Focus on Distribution, Risk Assee ssment, and Sources. Toxics. 2022;10. Available: https://www.ncbi.nlm.nih.gov/pmc/articles/PMC9324633/pdf/toxics-10-00401.pdf 10.3390/toxics10070401 PMC9324633 35878306

[10] Abdel-Shafy HI, Mansour MSM. A review on polycyclic aromatic hydrocarbons: Source, environmental impact, effect on human health and remediation. Egypt J Pet. 2016;25: 107–123. doi: 10.1016/J.EJPE.2015.03.011

[11] Yu H. ENVIRONMENTAL CARCINOGENIC POLYCYCLIC AROMATIC HYDROCARBONS: PHOTOCHEMISTRY AND PHOTOTOXICITY. J Environ Sci Health C Environ Carcinog Ecotoxicol Rev. 2002;20: 149–183. doi: 10.1081/GNC-120016203 12515673 PMC3812823

[12] Lv J, Xu J, Guo C, Zhang Y, Bai Y, Meng W. Spatial and temporal distribution of polycyclic aromatic hydrocarbons (PAHs) in surface water from Liaohe River Basin, northeast China. Environ Sci Pollut Res Int. 2014;21: 7088–7096. doi: 10.1007/s11356-014-2604-6 24554296

[13] Tang Z, Guo J, Liao H, Zhao X, Wu F, Zhu Y, et al. Spatial and temporal distribution and sources of polycyclic aromatic hydrocarbons in sediments of Taihu Lake, eastern China. Environ Sci Pollut Res. 2015;22: 5350–5358. doi: 10.1007/s11356-014-3746-2 25354436

[14] White KL. An overview of immunotoxicology and carcinogenic polycyclic aromatic hydrocarbons. Environ Carcinog Rev. 2008;4: 163–202. doi: 10.1080/10590508609373342

[15] Baird WM, Hooven LA, Mahadevan B. Carcinogenic polycyclic aromatic hydrocarbon-DNA adducts and mechanism of action. Environ Mol Mutagen. 2005;45: 106–114. doi: 10.1002/em.20095 15688365

[16] Feng J, Zhai M, Sun J, Liu Q. Distribution and sources of polycyclic aromatic hydrocarbons (PAHs) in sediment from the upper reach of Huaihe River, East China. Environ Sci Pollut Res Int. 2012;19: 1097–1106. doi: 10.1007/s11356-011-0620-3 21948143

[17] Qiu YW, Zhang G, Liu GQ, Guo LL, Li XD, Wai O. Polycyclic aromatic hydrocarbons (PAHs) in the water column and sediment core of Deep Bay, South China. Estuar Coast Shelf Sci. 2009;83: 60–66. doi: 10.1016/J.ECSS.2009.03.018

[18] Perra G, Renzi M, Guerranti C, Focardi SE. Polycyclic aromatic hydrocarbons pollution in sediments: Distribution and sources in a lagoon system (Orbetello, Central Italy). Transitional Waters Bull. 2009;3: 45–58. doi: 10.1285/i1825229Xv3n1p45

[19] Simpson Christopher D., Harrington Christopher F. and, Cullen* WR, and DAB, Reimer‡ KJ. Polycyclic Aromatic Hydrocarbon Contamination in Marine Sediments near Kitimat, British Columbia. Environ Sci Technol. 1998;32: 3266–3272. doi: 10.1021/ES970419Y

[20] Helfrich J, Armstrong DE. Polycyclic Aromatic Hydrocarbons in Sediments of the Southern Basin of Lake Michigan. J Great Lakes Res. 1986;12: 192–199. doi: 10.1016/S0380-1330(86)71718-8

[21] Yan W, Chi J, Wang Z, Huang W, Zhang G. Spatial and temporal distribution of polycyclic aromatic hydrocarbons (PAHs) in sediments from Daya Bay, South China. Environ Pollut. 2009;157: 1823–1830. doi: 10.1016/j.envpol.2009.01.023 19251344

[22] Ladwani KD, Ladwani KD, Ramteke DS. Assessment of poly aromatic hydrocarbon (PAH) dispersion in the near shore environment of Mumbai, India after a large scale oil spill. Bull Environ Contam Toxicol. 2013;90: 515–520. doi: 10.1007/s00128-012-0955-6 23412696

[23] Koyama J, Uno S, Nagai Y, Anukorn B. Early monitoring of spilled oil contamination in Rayong, Thailand. 環境毒性学会誌. 2016;19: 25–33. doi: 10.11403/JSET.19.25

[24] Koyama J, Uno S, Kohno K. Polycyclic aromatic hydrocarbon contamination and recovery characteristics in some organisms after the Nakhodka oil spill. Mar Pollut Bull. 2004;49: 1054–1061. doi: 10.1016/j.marpolbul.2004.07.010 15556192

[25] Uno S, Kokushi E, Añasco NC, Iwai T, Ito K, Koyama J. Oil spill off the coast of Guimaras Island, Philippines: Distributions and changes of polycyclic aromatic hydrocarbons in shellfish. Mar Pollut Bull. 2017;124: 962–973. doi: 10.1016/j.marpolbul.2017.03.062 28400055

[26] McNutt MK, Camilli R, Crone TJ, Guthrie GD, Hsieh PA, Ryerson TB, et al. Review of flow rate estimates of the Deepwater Horizon oil spill. Proc Natl Acad Sci U S A. 2012;109: 20260–20267. doi: 10.1073/pnas.1112139108 22187459 PMC3528583

[27] Arora MP, Lodhia S. The BP Gulf of Mexico oil spill: Exploring the link between social and environmental disclosures and reputation risk management. J Clean Prod. 2017;140: 1287–1297. doi: 10.1016/J.JCLEPRO.2016.10.027

[28] Murphy D, Gemmell B, Vaccari L, Li C, Bacosa H, Evans M, et al. An in-depth survey of the oil spill literature since 1968: Long term trends and changes since Deepwater Horizon. Mar Pollut Bull. 2016;113: 371–379. doi: 10.1016/j.marpolbul.2016.10.028 27773534

[29] Romo-Curiel AE, Ramírez-Mendoza Z, Fajardo-Yamamoto A, Ramírez-León MR, García-Aguilar MC, Herzka SZ, et al. Assessing the exposure risk of large pelagic fish to oil spills scenarios in the deep waters of the Gulf of Mexico. Mar Pollut Bull. 2022;176: 113434. doi: 10.1016/j.marpolbul.2022.113434 35183025

[30] Magalhães KM, Carreira RS, Rosa Filho JS, Rocha PP, Santana FM, Yogui GT. Polycyclic aromatic hydrocarbons (PAHs) in fishery resources affected by the 2019 oil spill in Brazil: Short-term environmental health and seafood safety. Mar Pollut Bull. 2022;175: 113334. doi: 10.1016/j.marpolbul.2022.113334 35091343

[31] Wang HS, Liang P, Kang Y, Shao DD, Zheng GJ, Wu SC, et al. Enrichment of polycyclic aromatic hydrocarbons (PAHs) in mariculture sediments of Hong Kong. Environ Pollut. 2010;158: 3298–3308. doi: 10.1016/j.envpol.2010.07.022 20708314

[32] Yaghmour F, Els J, Maio E, Whittington-Jones B, Samara F, El Sayed Y, et al. Oil spill causes mass mortality of sea snakes in the Gulf of Oman. Sci Total Environ. 2022;825: 154072. doi: 10.1016/j.scitotenv.2022.154072 35217042

[33] O’Sullivan AJ. The Amoco Cadiz oil spill. Mar Pollut Bull. 1978;9: 123–128. doi: 10.1016/0025-326X(78)90586-6

[34] Bellier P, Massart G. THE AMOCO CADIZ OIL SPILL CLEANUP OPERATIONS–AN OVERVIEW OF THE ORGANIZATION, CONTROL, AND EVALUATION OF THE CLEANUP TECHNIQUES EMPLOYED. Int Oil Spill Conf Proc. 1979;1979: 141–146. doi: 10.7901/2169-3358-1979-1-141

[35] Stalcup D, Yoshioka G, Black E, Carpenter M. Comparing oil spill rates using different data sources. 2005 Int Oil Spill Conf IOSC 2005. 2005; 10246–10259. doi: 10.7901/2169-3358-2003-1-861

[36] Adams JE, Brown RS, Hodson P V. The bioavailability of oil droplets trapped in river gravel by hyporheic flows. Environ Pollut. 2021;269: 116110. doi: 10.1016/j.envpol.2020.116110 33310493

[37] Lee K, Boufadel M, Chen B, Foght J, Hodson P, Swanson S, et al. Expert Panel Report on the Behaviour and Environmental Impacts of Crude Oil Released into Aqueous Environments. Royal Society of Canada. 2015. Available: http://www.rsc.ca/sites/default/files/pdf/OIW_Executive%20Summary_1.pdf%0Ahttps://www.rsc-src.ca/sites/default/files/pdf/OIWReport.pdf

[38] Jeznach LC, Mohan A, Tobiason JE, Reckhow DA. Modeling Crude Oil Fate and Transport in Freshwater. Environ Model Assess 2020 261. 2020;26: 77–87. doi: 10.1007/S10666-020-09728-4

[39] Neff JM. Composition and Fate of Petroleum and Spill-Treating Agents in the Marine Environment. Sea Mamm Oil Confronting Risks. 1990; 1–33. doi: 10.1016/B978-0-12-280600-1.50006–4

[40] Ylitalo GM, Collier TK, Anulacion BF, Juaire K, Boyer RH, da Silva DAM, et al. Determining oil and dispersant exposure in sea turtles from the northern Gulf of Mexico resulting from the Deepwater Horizon oil spill. Endanger Species Res. 2017;33: 9–24. doi: 10.3354/ESR00762

[41] Yadava M. One Man Enquiry Committe report on Damages to Environment, Biodiversity, Wildlife, Forest & Ecology on account of BLOW OUT AND EXPLOSION AT OIL WELL No. BGN-5 Vol-1. 2021.

[42] Qureshi Q, Kolipakam V, Wakid A. Impact of oil well blowout at Baghjan oil field, Assam and resulting oil spill, on surrounding landscape. Dehradun; 2020.

[43] Dudhagara DR, Rajpara RK, Bhatt JK, Gosai HB, Sachaniya BK, Dave BP. Distribution, sources and ecological risk assessment of PAHs in historically contaminated surface sediments at Bhavnagar coast, Gujarat, India. Environ Pollut. 2016;213: 338–346. doi: 10.1016/J.ENVPOL.2016.02.030 26925756

[44] Rahmani AR, Islam MZ, Kasambe RM. Important bird areas in India: priority sites for conservation (revised and updated). Important Bird Biodivers Areas India Prior sites Conserv. 2016;1: 9.

[45] Choudhury A. The status of endangered species in northeast India. J Bombay Nat Hist Soc. 2006;103: 157–167. Available: https://www.researchgate.net/publication/312936127

[46] Subedi B, Balakrishna K, Sinha RK, Yamashita N, Balasubramanian VG, Kannan K. Mass loading and removal of pharmaceuticals and personal care products, including psychoactive and illicit drugs and artificial sweeteners, in five sewage treatment plants in India. J Environ Chem Eng. 2015;3: 2882–2891. doi: 10.1016/j.jece.2015.09.031

[47] Das P, Joshi S, Rout J, Upreti DK. Impact of anthropogenic factors on abundance variability among lichen species in southern Assam, north east India. Trop Ecol. 2013;54: 67–72.

[48] Tuvikene A. Responses of fish to polycyclic aromatic hydrocarbons (PAHs) on JSTOR. In: Ann. Zool. Fennici [Internet]. 1 Nov 1995 [cited 4 Jul 2022] pp. 295–309. Available: https://www.jstor.org/stable/23735700

[49] Beyer J, Jonsson G, Porte C, Krahn MM, Ariese F. Analytical methods for determining metabolites of polycyclic aromatic hydrocarbon (PAH) pollutants in fish bile: A review. Environ Toxicol Pharmacol. 2010;30: 224–244. doi: 10.1016/j.etap.2010.08.004 21787655

[50] Chen CF, Ju YR, Su YC, Lim YC, Kao CM, Chen CW, et al. Distribution, sources, and behavior of PAHs in estuarine water systems exemplified by Salt River, Taiwan. Mar Pollut Bull. 2020;154. doi: 10.1016/j.marpolbul.2020.111029 32319888

[51] Oduntan AO, Tavengwa NT, Cukrowska E, Mhlanga SD, Chimuka L. QuEChERS Method Development for Bio-monitoring of low molecular weight polycyclic aromatic hydrocarbons in South African carp fish using hplc-fluorescence: an initial assessment. South African J Chem. 2016;69: 98–104. doi: 10.17159/0379-4350/2016/V69A12

[52] Recabarren-Villalón T, Ronda AC, Oliva AL, Cazorla AL, Marcovecchio JE, Arias AH. Seasonal distribution pattern and bioaccumulation of Polycyclic aromatic hydrocarbons (PAHs) in four bioindicator coastal fishes of Argentina. Environ Pollut. 2021;291. doi: 10.1016/j.envpol.2021.118125 34536644

[53] Noutsopoulos C, Koumaki E, Sarantopoulos V, Mamais D. Analytical and mathematical assessment of emerging pollutants fate in a river system. J Hazard Mater. 2019;364: 48–58. doi: 10.1016/j.jhazmat.2018.10.033 30339932

[54] Malik A, Singh KP, Mohan D, Patel DK. Distribution of polycyclic aromatic hydrocarbons in Gomti river system, India. Bull Environ Contam Toxicol. 2004;72: 1211–1218. doi: 10.1007/s00128-004-0372-6 15362451

[55] Chakraborty P, Sakthivel S, Kumar B, Kumar S, Mishra M, Verma VK, et al. Spatial distribution of persistent organic pollutants in the surface water of River Brahmaputra and River Ganga in India. Rev Environ Health. 2014;29: 45–48. doi: 10.1515/reveh-2014-0014 24659603

[56] Khuman SN, Chakraborty P, Cincinelli A, Snow D, Kumar B. Polycyclic aromatic hydrocarbons in surface waters and riverine sediments of the Hooghly and Brahmaputra Rivers in the Eastern and Northeastern India. Sci Total Environ. 2018;636: 751–760. doi: 10.1016/j.scitotenv.2018.04.109 29723840

[57] Brindha K, Elango L. PAHs contamination in groundwater from a part of metropolitan city, India: A study based on sampling over a 10-year period. Environ Earth Sci. 2014;71: 5113–5120. doi: 10.1007/S12665-013-2914-X

[58] Mitra S, Blanchi TS. A preliminary assessment of polycyclic aromatic hydrocarbon distributions in the lower Mississippi River and Gulf of Mexico. Mar Chem. 2003;82: 273–288. doi: 10.1016/S0304-4203(03)00074-4

[59] Shi Z, Tao S, Pan B, Fan W, He XC, Zuo Q, et al. Contamination of rivers in Tianjin, China by polycyclic aromatic hydrocarbons. Environ Pollut. 2005;134: 97–111. doi: 10.1016/j.envpol.2004.07.014 15572228

[60] Wang JZ, Nie YF, Luo XL, Zeng EY. Occurrence and phase distribution of polycyclic aromatic hydrocarbons in riverine runoff of the Pearl River Delta, China. Mar Pollut Bull. 2008;57: 767–774. doi: 10.1016/j.marpolbul.2008.01.007 18289609

[61] Wang Y, Zhang S, Cui W, Meng X, Tang X. Polycyclic aromatic hydrocarbons and organochlorine pesticides in surface water from the Yongding River basin, China: Seasonal distribution, source apportionment, and potential risk assessment. Sci Total Environ. 2018;618: 419–429. doi: 10.1016/j.scitotenv.2017.11.066 29136593

[62] Wu H, Sun B, Li J. Polycyclic Aromatic Hydrocarbons in Sediments/Soils of the Rapidly Urbanized Lower Reaches of the River Chaohu, China. Int J Environ Res Public Health. 2019;16. doi: 10.3390/IJERPH16132302 31261819 PMC6651651

[63] Lu XY, Zhang T, Fang HHP. Bacteria-mediated PAH degradation in soil and sediment. Appl Microbiol Biotechnol. 2011;89: 1357–1371. doi: 10.1007/s00253-010-3072-7 21210104

[64] Kuppusamy S, Thavamani P, Venkateswarlu K, Lee YB, Naidu R, Megharaj M. Remediation approaches for polycyclic aromatic hydrocarbons (PAHs) contaminated soils: Technological constraints, emerging trends and future directions. Chemosphere. 2017;168: 944–968. doi: 10.1016/j.chemosphere.2016.10.115 27823779

[65] Feng J, Yang Z, Niu J, Shen Z. Remobilization of polycyclic aromatic hydrocarbons during the resuspension of Yangtze River sediments using a particle entrainment simulator. Environ Pollut. 2007;149: 193–200. doi: 10.1016/j.envpol.2007.01.001 17321654

[66] Gundlach ER. Oil Spills. 2019; 1323–1327. doi: 10.1007/978-3-319-93806-6_233

[67] Zhao Z, Qin Z, Cao J, Xia L. Source and Ecological Risk Characteristics of PAHs in Sediments from Qinhuai River and Xuanwu Lake, Nanjing, China. J Chem. 2017;2017. doi: 10.1155/2017/3510796

[68] Zuloaga O, Prieto A, Ahmed K, Sarkar SK, Bhattacharya A, Chatterjee M, et al. Distribution of polycyclic aromatic hydrocarbons in recent sediments of Sundarban mangrove wetland of India and Bangladesh: a comparative approach. Environ Earth Sci 2012 682. 2012;68: 355–367. doi: 10.1007/S12665-012-1743-7

[69] Wu Y, Wang X, Ya M, Li Y, Hong H. Seasonal variation and spatial transport of polycyclic aromatic hydrocarbons in water of the subtropical Jiulong River watershed and estuary, Southeast China. Chemosphere. 2019;234: 215–223. doi: 10.1016/j.chemosphere.2019.06.067 31220655

[70] Zeng X., Liu J, He L., Yu Z., Sheng G., Fu J. Occurrence of organic pollutant in sediments from several river mouths in western part of Taihu Lake and their potential environmental significance. Asian J Ecotoxicol. 2016;11: 465–472. Available: https://scholar.google.com/scholar_lookup?journal=Asian+J.+Ecotoxicol.&title=Occurrence+of+organic+pollutant+in+sediments+from+several+river+mouths+in+western+part+of+Taihu+Lake+and+their+potential+environmental+significance&author=X.Y.+Zeng&author=J.+Liu&author=L.X.+He&author=Z.Y.+Liu&author=Z.Q.+Yu&volume=11&publication_year=2016&pages=465-472&

[71] Yancheshmeh RA, Bakhtiari AR, Mortazavi S, Savabieasfahani M. Sediment PAH: Contrasting levels in the Caspian Sea and Anzali Wetland. Mar Pollut Bull. 2014;84: 391–400. doi: 10.1016/j.marpolbul.2014.05.001 24910181

[72] Macías-Zamora J V., Mendoza-Vega E, Villaescusa-Celaya JA. PAHs composition of surface marine sediments: a comparison to potential local sources in Todos Santos Bay, B.C., Mexico. Chemosphere. 2002;46: 459–468. doi: 10.1016/s0045-6535(01)00069-8 11829402

[73] Filho PJS, Luz LP da, Betemps GR, Caramão EB. Evaluation of surface sediment contamination by polycyclic aromatic hydrocarbons in the “Saco do Laranjal”—(Patos Lagoon, Brazil). Mar Pollut Bull. 2012;64: 1933–1937. doi: 10.1016/j.marpolbul.2012.04.010 22763282

[74] Inomata Y, Kajino M, Sato K, Ohara T, Kurokawa JI, Ueda H, et al. Emission and atmospheric transport of particulate PAHs in Northeast Asia. Environ Sci Technol. 2012;46: 4941–4949. doi: 10.1021/es300391w 22435795

[75] Tudoran MA, Putz M V. Polycyclic Aromatic Hydrocarbons: from In Cerebro to In Silico Eco-Toxicity Fate. Chem Bull “Politehnica” Univ Timisoara. 2012;57: 50–53. Available: http://www.chemicalbulletin.ro/admin/articole/86886art_11(50-53).pdf

[76] Malik A, Ojha P, Singh KP. Distribution of polycyclic aromatic hydrocarbons in edible fish from Gomti river, India. Bull Environ Contam Toxicol. 2008;80: 134–138. doi: 10.1007/s00128-007-9331-3 18183338

[77] Dhananjayan V, Muralidharan S. Polycyclic Aromatic Hydrocarbons in Various Species of Fishes from Mumbai Harbour, India, and Their Dietary Intake Concentration to Human. Int J Oceanogr. 2012;2012: 1–6. doi: 10.1155/2012/645178

[78] Akpambang VOE, Purcaro G, Lajide L, Amoo IA, Conte LS, Moret S. Determination of polycyclic aromatic hydrocarbons (PAHs) in commonly consumed Nigerian smoked/grilled fish and meat. Food Addit Contam—Part A Chem Anal Control Expo Risk Assess. 2009;26: 1096–1103. doi: 10.1080/02652030902855406 19680985

[79] Alomirah H, Zenki S Al Husain A, Ahmed N, Rashdan A Al, Gevao B, et al. Dietary exposure to polycyclic aromatic hydrocarbons from commercially important seafood of the Arabian Gulf. J Food, Agric Environ. 2009;7: 9–15. Available: https://www.wflpublisher.com/Abstract/1381

[80] Cheung KC, Leung HM, Kong KY, Wong MH. Residual levels of DDTs and PAHs in freshwater and marine fish from Hong Kong markets and their health risk assessment. Chemosphere. 2007;66: 460–468. doi: 10.1016/j.chemosphere.2006.06.008 16870232

[81] Mishra S, Chauhan G, Verma S, Singh U. The emergence of nanotechnology in mitigating petroleum oil spills. Mar Pollut Bull. 2022;178: 113609. doi: 10.1016/j.marpolbul.2022.113609 35417809

[82] Armstrong B, Hutchinson E, Unwin J, Fletcher T. Lung Cancer Risk after Exposure to Polycyclic Aromatic Hydrocarbons: A Review and Meta-Analysis. Environ Health Perspect. 2004;112: 970. doi: 10.1289/ehp.6895 15198916 PMC1247189

[83] Council C. © Canadian Council of Ministers of the Environment, 2010. Occupational Health. 2010.

[84] González-Gaya B, Martínez-Varela A, Vila-Costa M, Casal P, Cerro-Gálvez E, Berrojalbiz N, et al. Biodegradation as an important sink of aromatic hydrocarbons in the oceans. Nat Geosci 2019 122. 2019;12: 119–125. doi: 10.1038/s41561-018-0285-3

[85] Malan DE. Effects of Qatar light crude oil on adult saltmarsh crab Sesarma catenata and implications in the field: respiration, oxygen diffusion and hypoxia. African J Mar Sci. 2010;4: 265–275. doi: 10.2989/025776186784461648

[86] Whitehead A. Interactions between Oil-Spill Pollutants and Natural Stressors Can Compound Ecotoxicological Effects. Integr Comp Biol. 2013;53: 635. doi: 10.1093/icb/ict080 23842611 PMC3895973

[87] Beyer A, Biziuk M. Environmental fate and global distribution of polychlorinated biphenyls. Rev Environ Contam Toxicol. 2009;201: 137–158. doi: 10.1007/978-1-4419-0032-6_5 19484591

[88] Sverdrup LE, Nielsen T, Krogh PH. Soil Ecotoxicity of Polycyclic Aromatic Hydrocarbons in Relation to Soil Sorption, Lipophilicity, and Water Solubility. Environ Sci Technol. 2002;36: 2429–2435. doi: 10.1021/es010180s 12075800

[89] Peterson CH, Rice SD, Short JW, Esler D, Bodkin JL, Ballachey BE, et al. Long-Term Ecosystem Response to the Exxon Valdez Oil Spill. Science (80-). 2003;302: 2082–2086. doi: 10.1126/SCIENCE.1084282/SUPPL_FILE/PETERSON.SOM.PDF 14684812

[90] Kainz MJ, Fisk AT. Integrating lipids and contaminants in aquatic ecology and ecotoxicology. Lipids Aquat Ecosyst. 2009;9780387893662: 93–114. doi: 10.1007/978-0-387-89366-2_5/COVER/

[91] Bejarano AC, Michel J. Large-scale risk assessment of polycyclic aromatic hydrocarbons in shoreline sediments from Saudi Arabia: environmental legacy after twelve years of the Gulf war oil spill. Environ Pollut. 2010;158: 1561–1569. doi: 10.1016/j.envpol.2009.12.019 20092920

[92] Honda M, Suzuki N. Toxicities of Polycyclic Aromatic Hydrocarbons for Aquatic Animals. Int J Environ Res Public Health. 2020;17. doi: 10.3390/ijerph17041363 32093224 PMC7068426

[93] Neff J. Polycyclic aromatic hydrocarbons in the aquatic environment (Journal Article) | ETDEWEB. In: Journal Article [Internet]. 1980 [cited 4 Jul 2022]. Available: https://www.osti.gov/etdeweb/biblio/5162466

[94] Veltman K, Huijbregts MAJ, Rye H, Hertwich EG. Including impacts of particulate emissions on marine ecosystems in life cycle assessment: the case of offshore oil and gas production. Integr Environ Assess Manag. 2011;7: 678–686. doi: 10.1002/ieam.246 21735543

[95] Dong C Di Chen CF, Chen CW. Determination of polycyclic aromatic hydrocarbons in industrial harbor sediments by GC-MS. Int J Environ Res Public Health. 2012;9: 2175–2188. doi: 10.3390/ijerph9062175 22829797 PMC3397371

[96] Venn-Watson S, Colegrove KM, Litz J, Kinsel M, Terio K, Saliki J, et al. Adrenal Gland and Lung Lesions in Gulf of Mexico Common Bottlenose Dolphins (Tursiops truncatus) Found Dead following the Deepwater Horizon Oil Spill. PLoS One. 2015;10: e0126538. doi: 10.1371/journal.pone.0126538 25992681 PMC4439104

[97] Gentina T, Tillie-Leblond I, Birolleau S, Faycal S, Saelens T, Boudoux L, et al. Fire-eater’s lung: seventeen cases and a review of the literature. Medicine (Baltimore). 2001;80: 291–297. doi: 10.1097/00005792-200109000-00002 11552082

[98] Schwacke LH, Smith CR, Townsend FI, Wells RS, Hart LB, Balmer BC, et al. Health of common bottlenose dolphins (Tursiops truncatus) in Barataria Bay, Louisiana, following the deepwater horizon oil spill. Environ Sci Technol. 2014;48: 93–103. doi: 10.1021/es403610f 24350796

[99] De Guise S, Levin M, Gebhard E, Jasperse L, Hart LB, Smith CR, et al. Changes in immune functions in bottlenose dolphins in the northern Gulf of Mexico associated with the Deepwater Horizon oil spill. Endanger Species Res. 2017;33: 291–303. doi: 10.3354/ESR00814

[100] Ballachey BE, Bodkin JL, DeGange AR. An Overview of Sea Otter Studies. Mar Mamm Exxon Vald. 1994; 47–59. doi: 10.1016/B978-0-12-456160-1.50010–2

[101] Esler D, Bowman TD, Trust KA, Ballachey BE, Dean TA, Jewett SC, et al. Harlequin duck population recovery following the “Exxon Valdez” oil spill: Progress, process and constraints. Mar Ecol Prog Ser. 2002;241: 271–286. doi: 10.3354/MEPS241271

[102] Helm RC, Costa DP, DeBruyn TD, O’Shea TJ, Wells RS, Williams TM. Overview of Effects of Oil Spills on Marine Mammals. Handb Oil Spill Sci Technol. 2015; 455–475. doi: 10.1002/9781118989982.CH18

[103] Schwacke LH, Thomas L, Wells RS, McFee WE, Hohn AA, Mullin KD, et al. Quantifying injury to common bottlenose dolphins from the Deepwater Horizon oil spill using an age-, sex- and class-structured population model. Endanger Species Res. 2017;33: 265–279. doi: 10.3354/ESR00777

[104] Geraci JR. Physiologic and Toxic Effects on Cetaceans. Sea Mamm Oil Confronting Risks. 1990; 167–197. doi: 10.1016/B978-0-12-280600-1.50011–8

[105] Ramírez-León MR, Sosa-Nishizaki O, Pérez-Brunius P, Romo-Curiel AE, Ramírez-Mendoza Z, Fajardo-Yamamoto A, et al. Semi-quantitative risk assessment of marine mammal oil exposure: A case study in the western Gulf of Mexico. Front Mar Sci. 2023;10: 1034647. doi: 10.3389/FMARS.2023.1034647/BIBTEX

[106] Schreiber L, Fortin N, Mazza A, Maynard C, Wasserscheid J, Tremblay J, et al. An experimental oil spill at a tidal freshwater wetland along the St. Lawrence River re-visited after 21 years. Environ Res. 2023;216: 114456. doi: 10.1016/j.envres.2022.114456 36181891

[107] Hester MW, Willis JM, Baker MC. Oil Spills in Coastal Wetlands. Encycl Anthr. 2018;1–5: 67–76. doi: 10.1016/B978-0-12-809665-9.09896–7

[108] Zheng X, Wang H, Tao Y, Kou X, He C, Wang Z. Community diversity of soil meso-fauna indicates the impacts of oil exploitation on wetlands. Ecol Indic. 2022;144: 109451. doi: 10.1016/J.ECOLIND.2022.109451

