# Supplementary material for: Polycyclic Aromatic Hydrocarbons (PAHs) in aquatic ecosystem exposed to the 2020 Baghjan oil spill in upper Assam, India: Short-term toxicity and ecological risk assessment
Source: PLoS One. 2023 Nov 29;18(11):e0293601. doi: 10.1371/journal.pone.0293601 (PMC10686499; doi:10.1371/journal.pone.0293601)
Supplement: S1 Table — (DOCX) [file pone.0293601.s001.docx]

**S1 Table – The chemical structures of PAHs compounds and their physicochemical properties**

| **Compound** | **Log K_ow_** | **pKa** | **Water**  **solubility (mg/L)** | **MW**  **g/mol** | **Chemical**  **Formula** | **CAS** | **Structure** |
| --- | --- | --- | --- | --- | --- | --- | --- |
| Naphthalene (NaP) | 3.30 | 4.5 | 31.6 | 128.17 | C_10_H_8_ | 91-20-3 | 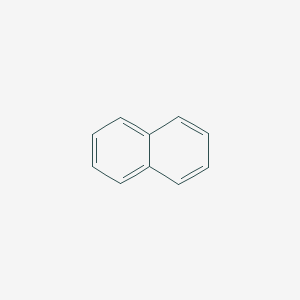 |
| Acenaphthylene (Acpy) | 3.93 | 4.45 | 1.5 | 152.19 | C_12_H_8_ | 208-96-8 | 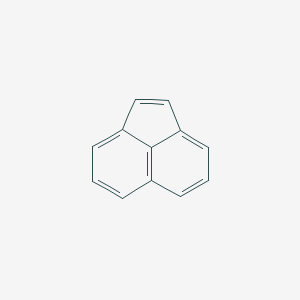 |
| Acenaphthene (Acp) | 3.92 |  | 4.0 | 154.21 | C_12_H_10_ | 83-32-9 | 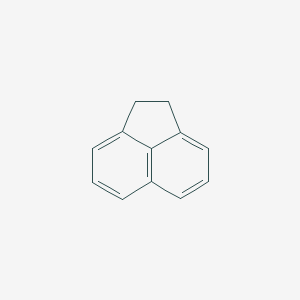 |
| Fluorene (Fl) | 4.18 | 17.79 | 2.5 | 166.22 | C_13_H_10_ | 86-73-7 | 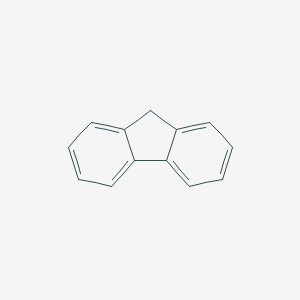 |
| Phenanthrene (Phe) | 4.46 | 9.75 | 1.6 | 178.23 | C_14_H_10_ | 85-01-8 | 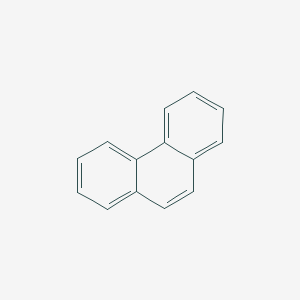 |
| Anthracene (Ant) | 4.45 | >15 | 0.044 | 178.23 | C_14_H_10_ | 120-12-7 | 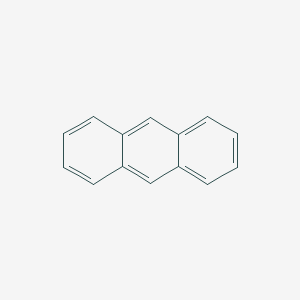 |
| Fluoranthene (Flu) | 5.16 |  | 0.265 | 202.25 | C_16_H_10_ | 206-44-0 | 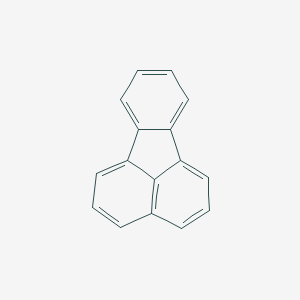 |
| Pyrene (Pyr) | 4.88 | -3.6 | 0.135 | 202.25 | C_16_H_10_ | 129-00-0 | 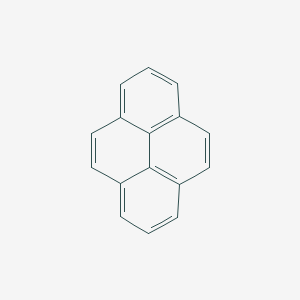 |
| Benz[a]anthracene (BaA) | 5.76 |  | 0.009 | 228.3 | C_18_H_12_ | 56-55-3 | 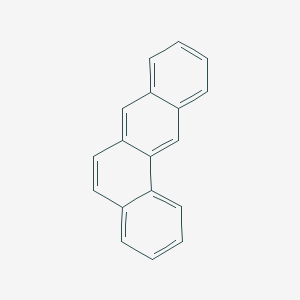 |
| Chrysene (Chr) | 5.73 |  | 0.002 | 228.3 | C_18_H0_12_ | 218-01-9 | 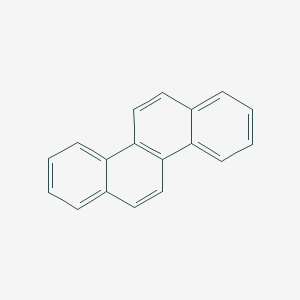 |
| Benzo[b]fluoranthene (BbF) | 5.78 |  | 0.0015 | 252.3 | C_20_H_12_ | 205-99-2 | 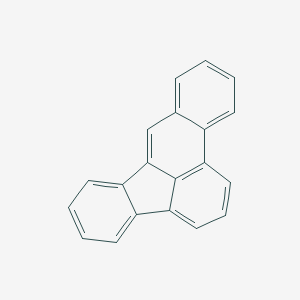 |
| Benzo[k]fluoranthene (BkF) | 6.11 |  | 0.0008 | 252.3 | C_20_H_12_ | 207-08-9 | 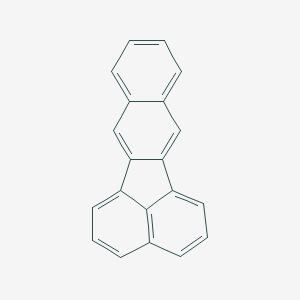 |
| Benzo[a]pyrene (BaP) | 6.13 | Strong acidic = 12.8, strong base = -3.6 | 0.0016 | 252.3 | C_20_H_12_ | 50-32-8 | 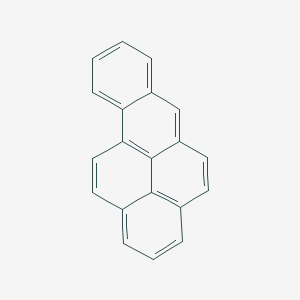 |
| Dibenz[a,h]anthracene (DbA) | 6.50 |  | 0.0024 | 278.3 | C_22_H_12_ | 57-70-3 | 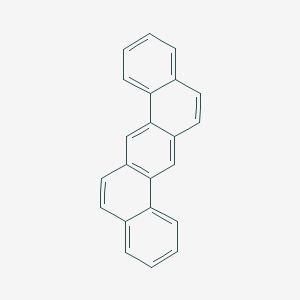 |
| Benzo[g,h,i]perylene (BghiP) | 6.63 |  | 0.0026 | 276.3 | C_22_H_12_ | 191-24-2 | 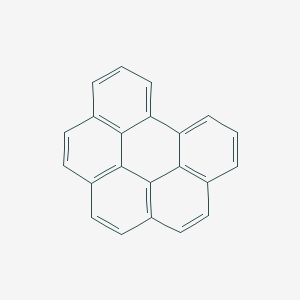 |
| Indeno[1,2,3-cd]pyrene (InP) | 6.70 |  | 0.0001 | 276.3 | C_22_H_12_ | 193-39-5 | 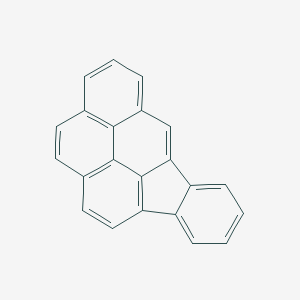 |
