# Supplementary material for: Polycyclic Aromatic Hydrocarbons (PAHs) in aquatic ecosystem exposed to the 2020 Baghjan oil spill in upper Assam, India: Short-term toxicity and ecological risk assessment
Source: PLoS One. 2023 Nov 29;18(11):e0293601. doi: 10.1371/journal.pone.0293601 (PMC10686499; doi:10.1371/journal.pone.0293601)
Supplement: S2 Table — (DOCX) [file pone.0293601.s002.docx]

**S2 Table - Details of Sampling locations**

| **Sampling Points** | **Latitude** | **Longitude** |
| --- | --- | --- |
| Sampling Point -1 | 27.59335 | 95.33629 |
| Sampling Point -2 | 27.58988 | 95.36539 |
| Sampling Point -3 | 27.60986 | 95.39125 |
| Sampling Point -4 | 27.60028 | 95.37898 |
| Sampling Point -5 | 27.57834 | 95.32655 |
| Sampling Point -6 | 27.58436 | 95.27390 |
| Sampling Point -7 | 27.57364 | 95.30464 |
| Sampling Point -8 | 27.59209 | 95.37782 |
| Sampling Point -9 | 27.57249 | 95.36883 |
| Sampling Point -10 | 27.58166 | 95.37909 |
| Sampling Point -11 | 27.58013 | 95.36620 |
| Sampling Point -12 | 27.57679 | 95.34931 |
