# Supplementary material for: Polycyclic Aromatic Hydrocarbons (PAHs) in aquatic ecosystem exposed to the 2020 Baghjan oil spill in upper Assam, India: Short-term toxicity and ecological risk assessment
Source: PLoS One. 2023 Nov 29;18(11):e0293601. doi: 10.1371/journal.pone.0293601 (PMC10686499; doi:10.1371/journal.pone.0293601)
Supplement: S3 Table — (DOCX) [file pone.0293601.s003.docx]

**S3 Table – Regression equations and coefficients of determination (R2) obtained for studied PAHs**

| **S. No** | **Compound** | **Regression Equation** | **Coefficients of**  **Determination (R2)** |
| --- | --- | --- | --- |
| **1** | Naphthalene (NaP) | y = 3391.3x + 35650.5 | 0.999 |
| **2** | Acenaphthylene (Acpy) | y = 5153.2x + 17905.1 | 0.999 |
| **3** | Acenaphthene (Acp) | y = 10093.5x + 52000.6 | 0.999 |
| **4** | Fluorene (Fl) | y = 13355.3x + 51842.2 | 0.999 |
| **5** | Phenanthrene (Phe) | y = 4793.9x + 13425.4 | 0.999 |
| **6** | Anthracene (Ant) | y = 3776.5x + 8351.4 | 0.999 |
| **7** | Fluoranthene (Flu) | y = 6516.7x + 11689.1 | 0.999 |
| **8** | Pyrene (Pyr) | y = 7639.02x + 16068.04 | 0.999 |
| **9** | Benz[a]anthracene (BaA) | y = 8107.6x - 74222.4 | 0.999 |
| **10** | Chrysene (Chr) | y = 8678.7x – 9494.6 | 0.999 |
| **11** | Benzo[b]fluoranthene (BbF) | y =4564.2x – 63066.5 | 0.998 |
| **12** | Benzo[k]fluoranthene (BkF) | y = 4586.2x – 21272.5 | 0.999 |
| **13** | Benzo[a]pyrene (BaP) | y = 3524.3x – 41106.5 | 0.998 |
| **14** | Indeno[1,2,3-cd]pyrene (InP) | y = 2599.1x -45573.4 | 0.997 |
| **15** | Dibenz[a,h]anthracene (DbA) | y = 1801.1x – 31228.3 | 0.997 |
| **16** | Benzo[g,h,i]perylene (BghiP) | y = 2823.7x – 32074.2 | 0.999 |
