# Supplementary material for: Polycyclic Aromatic Hydrocarbons (PAHs) in aquatic ecosystem exposed to the 2020 Baghjan oil spill in upper Assam, India: Short-term toxicity and ecological risk assessment
Source: PLoS One. 2023 Nov 29;18(11):e0293601. doi: 10.1371/journal.pone.0293601 (PMC10686499; doi:10.1371/journal.pone.0293601)
Supplement: S4 Table — (DOCX) [file pone.0293601.s004.docx]

**S4 Table – Molecular weights, quantification ion, confirmation ions and retention times of studied PAHs.**

| **S. No** | **Compound** | **Molecular Weight (g/mol)** | **Quantification Ions (m/z)** | **Confirmation Ions (m/z)** | **Retention Time** |
| --- | --- | --- | --- | --- | --- |
| **1** | Naphthalene (NaP) | 128.17 | 128 | 128.10, 102.00 | 8.1 |
| **2** | Acenaphthylene (Acpy) | 152.19 | 152 | 152.10, 151.10 | 11.8 |
| **3** | Acenaphthene (Acp) | 154.21 | 153 | 153.10, 152.10 | 12.3 |
| **4** | Fluorene (Fl) | 166.22 | 166 | 166.10, 165.10 | 14.0 |
| **5** | Phenanthrene (Phe) | 178.23 | 178 | 178.10, 152.10 | 17.8 |
| **6** | Anthracene (Ant) | 178.23 | 178 | 178.10, 152.10 | 18.0 |
| **7** | Fluoranthene (Flu) | 202.25 | 202 | 202.10, 199.90 | 23.0 |
| **8** | Pyrene (Pyr) | 202.25 | 202 | 202.10, 199.90 | 24.0 |
| **9** | Benz[a]anthracene (BaA) | 228.3 | 228 | 228.10, 226.00 | 29.6 |
| **10** | Chrysene (Chr) | 228.3 | 228 | 228.10, 226.00 | 29.8 |
| **11** | Benzo[b]fluoranthene (BbF) | 252.3 | 252 | 252.10, 249.90 | 34.4 |
| **12** | Benzo[k]fluoranthene (BkF) | 252.3 | 252 | 252.10, 249.90 | 34.5 |
| **13** | Benzo[a]pyrene (BaP) | 252.3 | 252 | 252.10, 249.90 | 35.7 |
| **14** | Indeno[1,2,3-cd]pyrene (InP) | 276.3 | 276 | 276.10, 274.00 | 41.1 |
| **15** | Dibenz[a,h]anthracene (DbA) | 278.3 | 278 | 278.10, 275.90 | 41.2 |
| **16** | Benzo[g,h,i]perylene (BghiP) | 276.3 | 276 | 276.10, 274.00 | 42.6 |
