# Supplementary material for: Polycyclic Aromatic Hydrocarbons (PAHs) in aquatic ecosystem exposed to the 2020 Baghjan oil spill in upper Assam, India: Short-term toxicity and ecological risk assessment
Source: PLoS One. 2023 Nov 29;18(11):e0293601. doi: 10.1371/journal.pone.0293601 (PMC10686499; doi:10.1371/journal.pone.0293601)
Supplement: S5 Table — (DOCX) [file pone.0293601.s005.docx]

**S5 Table - Dissolved Oxygen concentration at sampling sites**

| **Sampling Points** | **Dissolved Oxygen mg L^-1^** |
| --- | --- |
| Sampling Point -1 | 7.41 ± 0.18 |
| Sampling Point -2 | 7.14 ± 0.17 |
| Sampling Point -3 (Control Site) | 7.35 ± 0.09 |
| Sampling Point -4 | 7.11 ± 0.01 |
| Sampling Point -5 | 7.15 ± 0.07 |
| Sampling Point -6 | 7.24 ± 0.04 |
| Sampling Point -7 | 6.92 ± 0.14 |
| Sampling Point -8 | 0.93 ± 0.30 |
| Sampling Point -9 | 3.70 ± 0.25 |
| Sampling Point -10 | 1.06 ± 0.70 |
| Sampling Point -11 | 3.80 ± 0.58 |
| Sampling Point -12 | 5.29 ± 0.15 |
